# Glycerol Adsorption on TiO_2_ Surfaces: A Systematic Periodic DFT Study

**DOI:** 10.1002/open.202400153

**Published:** 2025-01-28

**Authors:** Andrés Camilo Muñoz Peña, Elizabeth Flórez, Francisco Núñez‐Zarur

**Affiliations:** ^1^ Chemistry and Biochemistry Department New Mexico State University 88001 Las Cruces, NM USA; ^2^ Facultad de Ciencias Básicas Universidad de Medellín 050026 Medellín Colombia; ^3^ Departamento de Química Facultad de Ciencias Universidad Nacional de Colombia − Sede Bogotá Carrera 30 No., 45–03 111321 Bogotá Colombia

**Keywords:** *ab initio* calculation, Adsorption energy, Glycerol adsorption, Titanium dioxide, TiO_2_

## Abstract

Conversion of glycerol to added‐value products is desirable due to its surplus during biodiesel synthesis. TiO_2_ has been the most explored catalyst. We performed a systematic study of glycerol adsorption on anatase (101), anatase (001), and rutile (110) TiO_2_ at the Density Functional Theory level. We found several adsorption modes on these surfaces, with anatase (101) being the less reactive one, leading to adsorption energies between −0.8 and −0.4 eV, with all adsorptions molecular in nature. On the contrary, anatase (001) is the most reactive surface, leading to both molecular and dissociative adsorption modes, with energies ranging from −4 to −1 eV and undergoing severe surface reconstructions in some cases. Rutile (110) also shows both molecular and dissociative adsorptions, but it is less reactive than anatase (001). Surfaces with oxygen vacancies affects the adsorbed states and energies. The electronic structure analysis reveals that glycerol adsorption mainly affects the band gap of the material and not the individual contributions to the valence and conduction band. Bader charge analysis shows that strong adsorption modes on anatase (001) and rutile (110) are associated with large charge transfer from glycerol to the surface, while weak and molecular adsorption modes involve low charge transfer.

## Graphical abstract

Extensive periodic DFT calculations reveals that anatase (001) is the most reactive surface for glycerol adsorption, which gives mostly dissociative modes, followed by rutile (110) and anatase (101) that mostly lead to molecular adsorption modes. Charge transfer was identified as the key descriptor to understand the adsorption process.

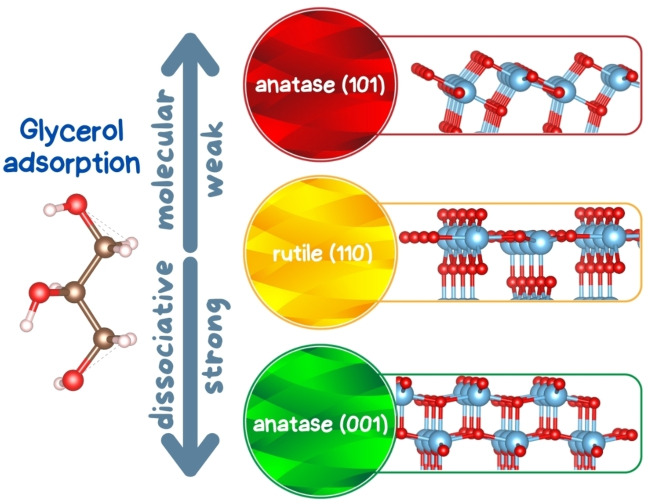

## Introduction

**Scheme 1:**
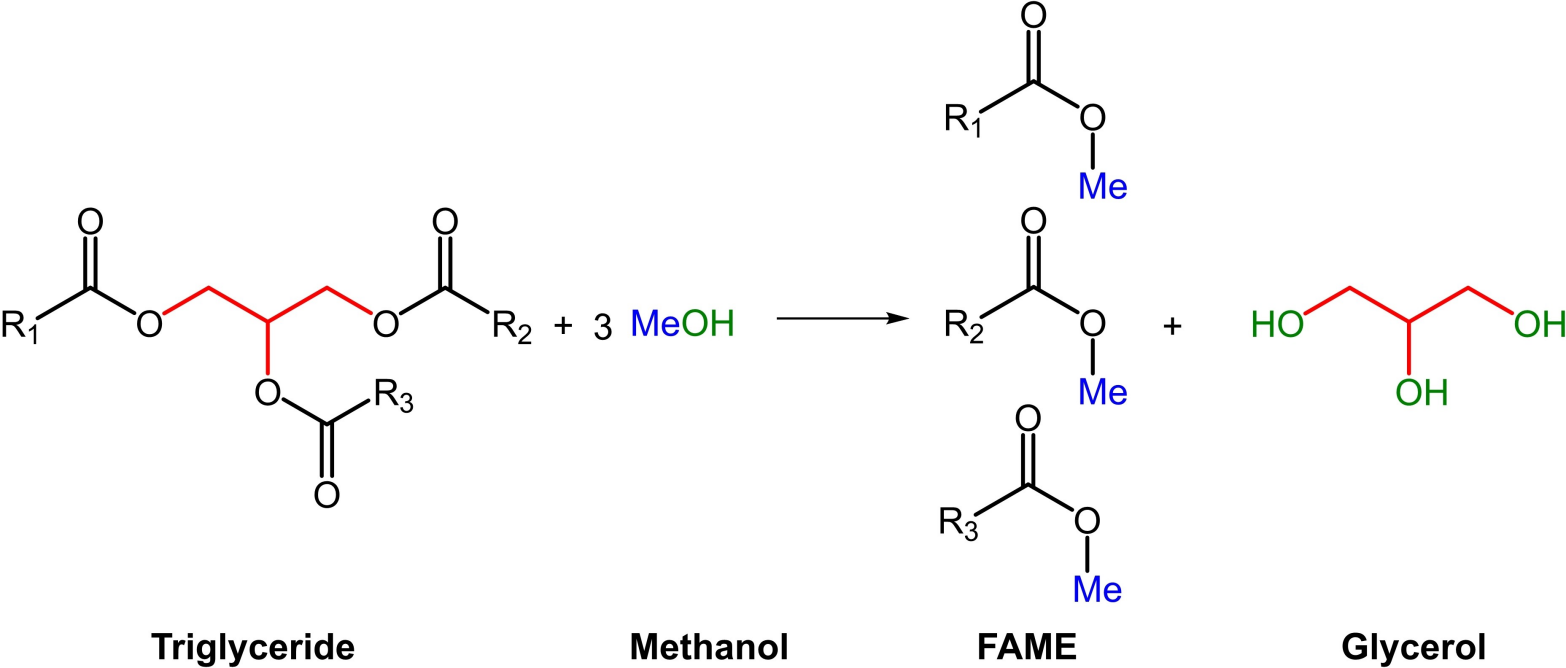
Transesterification of triglycerides to produce FAME and glycerol.

In the last years an important environmental concern has emerged due to the incoming energy crisis caused by the depletion of fossil fuels, which are the primary energy source that keeps our society moving forward. The intensive use of oil, coal, and natural gas as energy sources has released enormous amounts of CO_2_ into the atmosphere, with serious consequences to the environment and human health such as global warming and climate change. Fossil fuels also provide about 80 % of about 450 million tons of carbon source for the synthesis of chemical products of daily use. Thus, the chemical industry also contributes to ~7 % of global greenhouse gas emissions, such as CO_2_.^[1]^ Therefore, current research is focused on looking for renewable energy sources. Among several alternatives that include solar, wind, and geothermal, biomass was found one of the more sustainable ones.[^2^, ^3^] In this context, biodiesel is a promising biofuel that has gained interest because is biodegradable, non‐toxic, and can be used in many modes of transportation.^[1]^ Biodiesel, consisting of a mixture of fatty acids methyl esters (FAME), is mostly obtained by base catalyzed transesterification reaction of triglycerides with methanol, as shown in Scheme 1.^[4]^

Glycerol is the main subproduct from biodiesel synthesis, constituting 10 wt. %.^[5]^ As a subproduct, crude glycerol (from biodiesel synthesis) has limited applications since it needs to be purified, an extremely costly process. Moreover, the overwhelming production of biodiesel has led to an oversupply of glycerol, thus lowering its cost in the market.^[6]^ This effect offers an alternative to transforming glycerol into added‐value chemicals with a variety of applications.[^1^, ^2^, ^6^, ^7^, ^8^, ^9^, ^10^, ^11^] Several of these conversions include thermal conversion to lactic acid,^[12]^ dehydrogenation to produce syngas,^[13]^ hydrogenolysis oriented to propanediol,^[14]^ electrocatalytic oxidation,^[15]^ hydrogen production via reforming,^[16]^ and photocatalysis.[^6^, ^10^] Photocatalysis of glycerol is regarded as a promising route due to the adoption of many principles of green chemistry, such as atom economy, less hazardous reactions, conditions, and reagents.^[17]^ Compared with the other thermocatalytic processes, photocatalysis is selective under mild conditions and may open new reaction pathways not accessible from conventional methodologies.^[10]^ Among different semiconductor materials used for the photocatalytic transformation of glycerol, titanium dioxide (TiO_2_)[^18^, ^19^] is one of the most used catalysts. Most photo‐oxidation reactions of glycerol have been carried out with TiO_2_ supported metal particles, including Pd,[^20^, ^21^] Pt,[^21^, ^22^, ^23^, ^24^] Au[^21^, ^25^] and Cu combination with other metals, forming bimetallic particles.[^26^, ^27^] Generally, it has been observed that the photocatalytic activity is enhanced by the presence of the active metal. However, other experiments have also explored the photo‐oxidation of glycerol at the laboratory scale over pure TiO_2_.[^23^, ^28^, ^29^] For instance, partial photooxidation of glycerol using anatase and rutile was investigated using two reactors, an annular photoreactor, and cylindrical photoreactor, the latter being more efficient.^[28]^ In the aqueous phase, four main products were detected (among other unknowns): 1,3‐dihydroxyacetone, glyceraldehyde, formic acid, and carbon dioxide, with an average global selectivity of *ca*. 45 % with respect to the converted glycerol (using the cylindrical photoreactor). According to the evolution of these products, all of them are produced simultaneously, indicating the occurrence of parallel reactions in a heterogeneous surface with multiple active sites. In a different study, the kinetics and mechanisms of photo‐oxidation of glycerol over TiO_2_ and Pt/TiO_2_ samples were investigated.^[23]^ In general, the Pt modified catalyst shows better performance and higher rates due to better separation of electron‐holes pairs and the promotion of cathodic half reactions. Acetol, acetaldehyde, methanol, and ethanol were the main products. These products are obtained by initial hydrogenolysis and dehydration of glycerol to propylene glycol, and glyceraldehyde, respectively, which also undergo dehydrogenation, decarbonylation and hydrogenation reactions to 2‐oxopropanol, acetaldehyde, ethanol acetone, ethylene glycol and methanol. Also, the effect of several parameters such as the presence of H_2_O_2_ as oxidizing species, light intensity and irradiation time were investigated in the photo‐oxidation of glycerol using TiO_2_.^[29]^ For all tests, the irradiation time was the most important parameter. The main products detected are dihydroxyacetone, glyceraldehyde, and glyceric acid with selectivity of *ca*. 24 %, 68 %, and 8 %, respectively.

In seeking fundamental insights into the specific chemical interactions of glycerol with TiO_2_ surfaces, we will focus here on pure TiO_2_, both pristine and defective. That is, regardless of the chemical reaction for the conversion of glycerol, one prerequisite for the reaction to take place is the adsorption of glycerol onto the TiO_2_ surface. The interaction between TiO_2_ and alcohols has been long studied from experiments[^30^, ^31^, ^32^] and computations,[^33^, ^34^, ^35^] and the chemistry and dynamics of such interactions are now well understood. Sadly, most of these studies at the experimental and computational level were done with small alcohols like methanol and ethanol, and very few studies have considered glycerol. For instance, Carchini and López evaluated the adsorption of mono‐, bi‐, and poly‐alcohols (including glycerol) over rutile (110) using DFT.^[35]^ They found both molecular and dissociative adsorptions with energies between −1.86 and −1.37 eV for glycerol. In another study, Chermahini and co‐workers studied the adsorption and dehydration of glycerol to acrolein over a model of anatase (100) at the DFT level.^[36]^ In this case, four molecular adsorptions were considered and a competition in adsorption energy between the terminal and central hydroxyl was identified. In a more recent combined experimental and computational study, Wang and co‐workers proposed the selective oxidation of glycerol on rutile and anatase crystals at mild conditions.^[37]^ They found that while rutile is prone to transform glycerol into formic acid, anatase performs reforming to CO. All these studies provided significant insights into the adsorption and the chemistry of glycerol adsorbed on TiO_2_. However, there is still a knowledge gap that prevents successful catalyst design for the conversion of glycerol. For instance, they all were performed on a particular TiO_2_ surface and considered limited modes of glycerol adsorption (molecular or dissociative). Thus, in this work, we propose an extensive and systematic study of the molecular and dissociative adsorption modes of glycerol over rutile (100), anatase (101), and anatase (001) surfaces at the DFT level using the Periodic Boundary Conditions (PBC) approach. Also, we will provide an electronic analysis of the adsorbed states that will help to understand the adsorption process.

## Methodology and Surface Models

All calculations were carried out at the Perdew‐Burke‐Ernzerhof (PBE)^[38]^ level of the Density Functional Theory level using VASP.[^39^, ^40^, ^41^, ^42^] The projector augmented wave (PAW) method was used to describe the inner electrons except for the *d* electrons in titanium, which were treated explicitly.^[43]^ Van der Waals dispersion corrections were employed with the D3 approximation^[44]^ due to the hydrogen bonding present in the systems. The Kohn‐Sham orbitals were described using plane waves with a cutoff energy of 450 eV, a value that proved to be suitable for TiO_2_ as reported in the literature.^[35]^ The presence of dipole moments on the surfaces was also accounted for during calculations using the dipole correction to the total energy in the *z* direction. A Hubbard correction (U) was employed to improve the description of *d* orbitals of Ti. In this case U=4.2 eV was used as described in literature.^[35]^ To obtain the optimized geometries, the criteria for energy convergence and gradients were set to 10^–6^ eV and 0.05 eV/Å., respectively.

**Figure 1:**
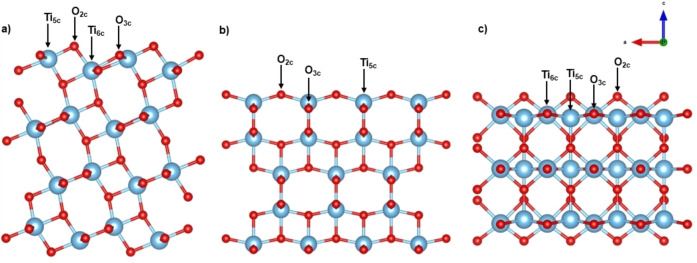
Optimized surface structures of a) A101; b) A001; c) R110. The most relevant exposed atoms on the surface are labeled.

In order to create the surface models of rutile (110), anatase (101), and anatase (001), we started from the bulk anatase and rutile TiO_2_ unit cells, which were obtained from the Materials Project database.^[45]^ Then, we fully optimized the cells as a function of the *k*‐point mesh to obtain converged *a*, *b*, and *c* parameters (for anatase *a* =3.803 Å; *b* =3.803 Å; *c* =9.487 Å; for rutile *a* =4.650 Å; *b* =4.650 Å; *c* =2.966 Å). These parameters at the PBE level show errors below 1 % compared to the experimental value, which indicates that this methodology is suitable for describing the lattice structures. For anatase and rutile unit cell relaxations *k*‐point grids of (8 8 3) and (8 8 13) were employed, respectively, as shown in Figure S1 of the Supporting Information (hereafter, SI). Then, surface models were created. To do this, 4x2 and 3x3 supercells of rutile and anatase, respectively, were created using VESTA.^[46]^ For rutile, the supercell was cut to expose the (110) facet and the resulting surface has 3 O−Ti‐O trilayers. In the case of anatase, two surfaces were created, (001) and (101), with 5 and 8 layers, respectively. In all cases, a 20 Å vacuum was added to the surfaces in the *z* direction to accommodate the incoming glycerol molecule and to avoid interaction with adjacent images (see Figure 1). For surface geometry optimizations, a Γ‐centered *k*‐point mesh of 3x3x1 was employed. The surfaces rutile (110), anatase (101), and anatase (001) will be named R110, A101 and A001 hereafter, respectively. During calculations of the clean slabs and the glycerol adsorption studies for R110, the 3 bottom layers were kept fixed during geometry optimizations while the upper 3 were allowed to relax. For A101, the 4 upper layers were optimized together with the glycerol while the bottom 4 layers were fixed. For A001, the 3 upper layers were optimized together with the glycerol while the bottom 2 layers were fixed. Since at reaction conditions (for instance, in the presence of water) the surface may suffer modifications, such as hydroxylation and point defects, in addition to the pristine surfaces we also tested glycerol adsorption modes on defective surfaces. The most common point defect in the photocatalytic reactivity of TiO_2_ materials is the oxygen vacancy.[^47^, ^48^, ^49^] Thus, for each surface investigated here, we created new models containing O vacancies by deleting one exposed O_2c_ atom, which is the most common site for vacancy creation (Figure S2 of the SI). This allows us to create an O vacancy defect and let the Ti atoms to be exposed to interact with the incoming glycerol molecule. This procedure is common for the modeling of O vacancy defects in A001,[^50^, ^51^] A101,[^52^, ^53^] and R110.^[54]^ Isolated glycerol was optimized using a 20x20x20 Å cubic box, with a Γ‐centered 1x1x1 *k*‐point mesh. Two different glycerol isomer structures were considered with an energy difference between them of *ca*. 0.11 eV (Figure S3 of SI), depending on the way the glycerol absorbs on the surface, mono‐ (C_gly‐1_) or bidentate (C_gly‐2_).

Adsorption of glycerol was studied for all three surfaces, considering both molecular and dissociative scenarios without bias. That is, glycerol was placed at different locations and distances from the TiO_2_ surfaces, and we let it freely evolve during geometry optimizations. The adsorption energy for glycerol is calculated as:

(1) $$\Delta E_{ads}=E_{TiO2+gly}-\left(E_{TiO2}+E_{gly}\right)\;\;\;$$

where Δ*E*_ads_ is the adsorption energy of glycerol, *E*_TiO2+gly_ is the total energy of the glycerol/surface system with one molecule adsorbed on the surface, *E*_TiO2_ is the total energy of clean TiO_2_ surface slab, and E_gly_ is the energy of the isolated glycerol molecule. We also calculated the Gibbs energy of glycerol adsorption onto the surfaces:

(2) $$\Delta G_{ads}=\Delta E_{ads}+\Delta E_{ZPE}-T\Delta S\;$$

where Δ*E*_ads_ is the electronic adsorption energy of glycerol as calculated by VASP, Δ*E*_ZPE_ is the zero‐point energy, T is the temperature and Δ*S* is the change in entropy during de adsorption process. The calculation of these quantities was done with VASPKIT.^[55]^ Total (TDOS) and Projected Density of States (PDOS) were performed to analyze the electronic structure of all surfaces before and after glycerol adsorption. Calculations were done with the same parameters as described above except for the *k*‐point mesh, which was adjusted to 6×6×1. Bader charge analysis using the Bader code from Henkelman and co‐workers[^56^, ^57^, ^58^, ^59^] was performed to better analyze charge transfer effects. The calculated Bader charges on the glycerol fragment in the adsorbed states is then plotted against the adsorption energies, and the linearity of these correlations is evaluated by the R^2^ parameter, calculated as:

(3) $$R^{2}=SSR/TSS$$

where SSR is the sum of squares due to regression and TSS is the total sum of squares about the mean, which is a measure of the variation between data points and the mean of the dataset.

## Results and Discussion

The results of the present paper will be presented in different sections. First, we will describe in detail all possible modes of the adsorption of glycerol on pristine (no defects) TiO_2_ model surfaces with energies based on electronic Konh‐Sham (KS) energies as reported by VASP as well as on Gibbs energies at room temperature. Second, we introduce a short comment on the role of surface defects in the adsorption of glycerol. Finally, an electronic structure analysis based on TDOS, PDOS, and Bader charge analysis is discussed.

### Glycerol Adsorption on A101

**Figure 2:**
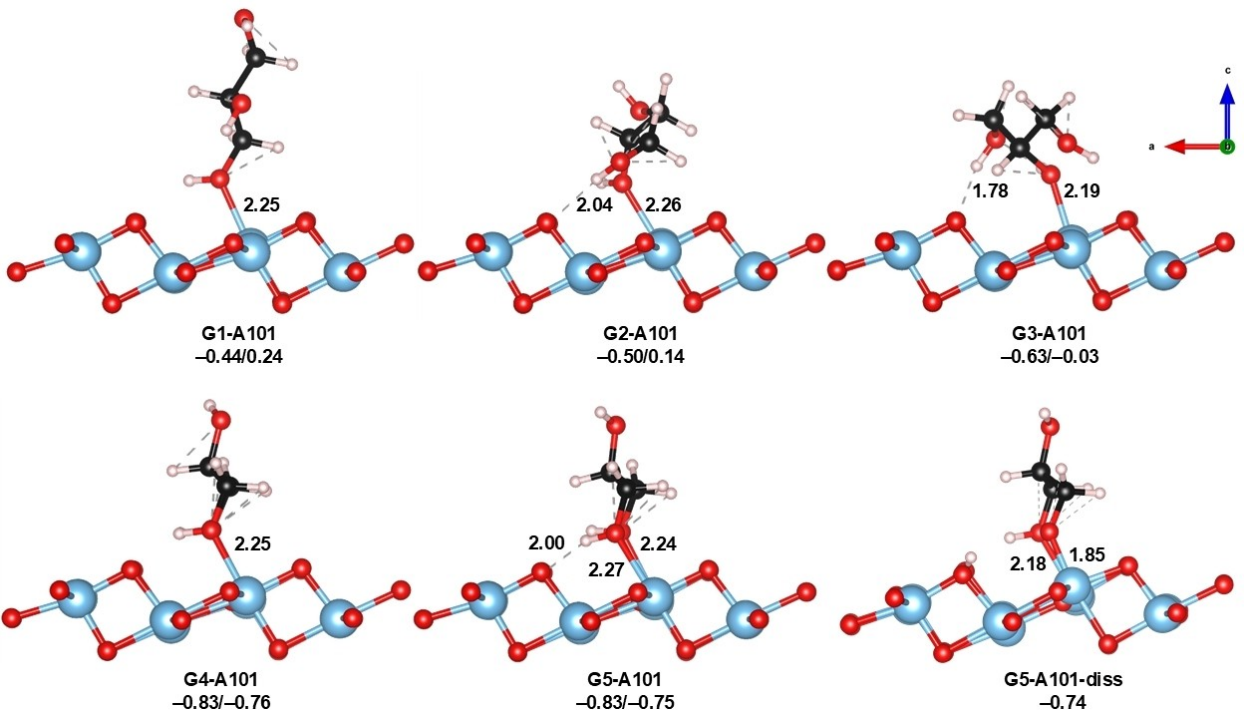
Optimized geometries of glycerol adsorbed on A101. Adsorption energies are also shown in terms of ΔE/ΔG_298_ (in eV). Distances are shown in Å.

A101 was investigated since it is the major facet exposed in natural anatase samples (along with the (001) surface)^[18]^ and has shown important applications, for instance, in catalysis.^[60]^ On this surface, both 5‐fold (Ti_5c_) and 6‐fold‐coordinated Ti atoms (Ti_6c_) are exposed, as well as 2‐fold (O_2c_) and 3‐fold‐coordinated O atoms (O_3c_), see Figure 1a. Thus, we were intrigued by the glycerol adsorption on all these sites. We found that Ti_5c_ and O_2c_ sites are the more reactive, which may interact with the hydroxyl groups of glycerol, leading to a set of five different adsorption modes that were identified after an extensive screening of several initial configurations. These modes are shown in Figure 2.

Calculations carried out here indicate that glycerol adsorption on Ti_5c_ is preferred over Ti_6c_, presumable because the latter is less geometrically accessible. Furthermore, on A101, all adsorption modes are molecular. The most favorable interaction in all cases involves the Ti_5c_ and the hydroxyl oxygen of glycerol, with Ti_5c_−O_gly_ distances in the range 2.19 to 2.27 Å. Adsorption modes G1‐G3 are monodentate, in which G2 and G3 correspond to interactions with the central hydroxyl of glycerol, while modes G4 and G5 involve geometrically similar bidentate configurations in which the terminal hydroxyls of glycerol interact with adjacent surface Ti_5c_. On this surface, glycerol adsorption shows a narrow range of energies, that is, between −0.83 and −0.44 eV. The upper limit adsorption corresponds to the monodentate mode G1, and the small differences in energy with the G2/G3 modes (0.05/0.18 eV) can be attributed to the formation of H‐bonding interactions between glycerol and the surface in the latter ones, which are not present in G1. Bidentate modes G4 and G5 show more favorable adsorption energies (−0.83 eV each) due to the double interaction between the terminal −OH's of glycerol and the surface. Since this is the only surface where all adsorption modes are molecular, we wanted to investigate whether forcing dissociation of glycerol would lead to more favorable configurations. To this end, from G5‐A101, we optimized a structure where one proton was transferred to the O_2c_ exposed on the surface, leading to a hydroxylated surface (G5‐A101‐diss). Although the surface was successfully located, the energy of this state is −0.74 eV, a very similar value to the non‐dissociated states G4 and G5 (Figure 2). This suggests that even by forcing the dissociation on this surface, the adsorption energy is invariant, emphasizing the idea that these exposed oxygen atoms in A101 are not basic enough to dissociate the −OH of glycerol. Thus, although dissociation is possible on this surface (although not spontaneous from free glycerol), this dissociation does not lead to more stable configurations.

The small values in adsorption energies for A101 agree with the stability of this surface reported in the literature.^[61]^ Few articles have discussed the adsorption of small alcohols on the A101 surface. For instance, Selloni and co‐workers reported the adsorption of methanol on A101 with similar adsorption energies as obtained in this work.^[62]^ Additionally, Tian and coworkers also observed the same behavior during the adsorption of 2‐propanol onto this surface.^[63]^ These results are in agreement with the results reported here and demonstrate that the A101 shows practically the same reactivity regardless of the nature of the alcohol. Finally, when considering Gibbs energies at 298 K, we observe that modes G1 and G2 become unfavorable, as the entropy loss of the adsorption process is not compensated by the chemical interaction. G3 is almost isoenergetic and G4/G5 are still favorable adsorption processes.

### Glycerol Adsorption on A001

**Figure 3:**
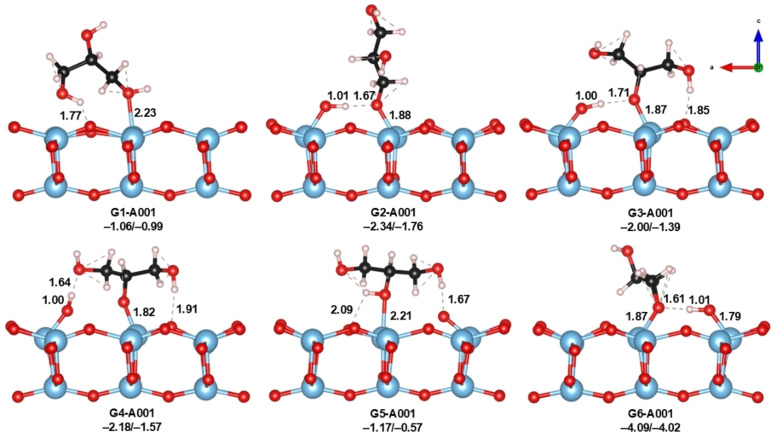
Optimized geometries of glycerol adsorbed on A001. Adsorption energies are also shown in terms of ΔE/ΔG_298_ (in eV). Distances are shown in Å.

The adsorption process on A001 is less reported experimentally due to the inherent difficulty of synthesizing pure A001 crystals. This surface has 0.46 J/m^2^ more energy compared to A101, indicating a more reactive surface compared to others in the same crystalline phase.^[64]^ Nevertheless, the adsorption of small molecules such as O_2_, H_2_O, CO_2_, NH_3_, formic acid, and others have been previously studied using DFT methods due to the importance of this surface in catalytic reactions.[^65^, ^66^, ^67^, ^68^] On the A001 surface 5‐fold Ti atoms (Ti_5c_) and 2‐fold/3‐fold‐coordinated O atoms (O_2c_/O_3c_) are exposed (see Figure 1b). In this case, 8 different initial configurations for the adsorption of glycerol were tested, which finally resulted in 6 adsorption modes, as shown in Figure 3. We found two molecular and four dissociative modes, which indicates that on this surface the adsorption of glycerol is accompanied by surface reconstruction. A general consideration worth mentioning is that the glycerol adsorption energies are much more favorable on A001 compared to A101. Even the molecular mode G1 shows higher adsorption energy than any mode on the A101, corroborating the high reactivity of this surface.

The molecular adsorption mode is G1, and no reconstruction of the surface is observed in this case. Here, a Ti_5c_−O_gly_ bond of 2.23 Å is formed through the terminal −OH of glycerol. A secondary H‐bond interaction between the other terminal −OH of glycerol and one vicinal O_2c_ is established, with a distance of 1.77 Å. This interaction pulls upward the O_2c_ slightly from the surface, but it is not strong enough to break the Ti_5c_−O_2c_ bond. The next three modes (G2‐G4) correspond to dissociative monodentate adsorptions. In G2, a Ti_5c_−O_gly_ bond is formed via the terminal −OH of glycerol. In this mode, glycerol is adsorbed almost perpendicular to the surface, and the reconstruction of the surface implies a proton transfer from the glycerol to one O_2c_ of the surface. The newly formed bonds Ti_5c_−O_gly_ and O_2c_−H show distances of 1.88 and 1.01 Å, respectively, with a secondary H‐bond interaction between the new hydroxyl and the O_gly_ of about 1.67 Å. Modes G3 and G4 represent adsorptions via central −OH of glycerol and are similar in geometry and energy. In these two modes, adsorption is accompanied by concomitant proton transfer from the central hydroxyl to the neighbor O_2c_ and a H‐bond interaction between one of the terminal −OH's and one O_2c_ on the surface (with distances of 1.85 and 1.91 Å for G3 and G4, respectively). The Ti_5c_−O_gly_ bond distances are 1.87 and 1.82 Å, respectively, while the new O−H distances features the same value in both configurations (1 Å). The main difference between G3 and G4 comes from the interaction of the new surface −OH with the glycerol molecule: while in G3 this is done with the central oxygen, in G4 it is established with the other terminal −OH of glycerol. There is also a molecular adsorption mode that involves surface reconstruction, the G5 mode. It is similar to G3 and G4 in that the adsorption takes place from the central −OH group of glycerol, but no glycerol dissociation is present and thus, it remains in its molecular form. The Ti_5c_−O_gly_ bond distance is 2.21 Å. However, one of the secondary H‐bond interactions between the terminal −OH group of glycerol and the O_2c_ on the surface is strong enough to break the Ti_5c_−O_2c_ bond. This interaction is characterized by a distance of 1.67 Å, much shorter than similar interactions in G3, G4 and the molecular mode G1. Thus, although glycerol is adsorbed in the molecular state, the closeness between glycerol and the surface induces surface reconstruction. Finally, we also located a dissociative model in which the two terminal −OH groups of glycerol interact with adjacent Ti_5c_. Thus, this state corresponds to a bidentate configuration, in which the formation of the two Ti_5c_−O_gly_ bonds (~1.87 Å) is accompanied by the dissociation of glycerol hydroxyls, generating a severe surface reconstruction. The newly formed surface hydroxyls interact with the O_gly_ at a distance of 1.61 Å.

The above results demonstrate the high reactivity of the A001 surface of TiO_2_. The fact that the main interactions between glycerol and the surface induce reconstruction by breaking Ti_5c_−O_2c_ bonds implies very favorable adsorption energies. Indeed, the calculated energies range from −4.09 (G6) to −1.06 eV (G1) and they roughly indicate the strength of the acid‐base interactions present in the glycerol adsorption process. For instance, the molecular mode with no bond breaking G1 shows the less favorable adsorption, while the double dissociated state G6 with severe surface reconstruction shows the most favorable one. In the middle, we found dissociative modes with modest surface reconstruction, such as G2, G3, and G4, with adsorption energies around −2 eV. The molecular mode G5 that induces reconstruction is associated with an adsorption energy of only −1.17 eV. Similar to the case of A101 presented above, the reactivity of A001 surfaces has not been largely explored at the computational level. However, few studies on methanol and 2‐propanol adsorption on A001 demonstrate that our results are consistent with the literature. Tian^[63]^ and Selloni^[69]^ independent reports on the adsorption of 2‐propanol and methanol showed that the alcohols dissociate upon adsorption on A001, even at low coverages, in contrast to A101, for which no dissociation is observed. The reported energies for 2‐propanol are also comparable with the ones found in this work for molecular and dissociative glycerol adsorption. It is worth mentioning here that even when the enthalpy and entropy corrections are considered, all adsorption modes remain highly favorable, suggesting the high reactivity of this surface.

### Glycerol adsorption on R110

**Figure 4:**
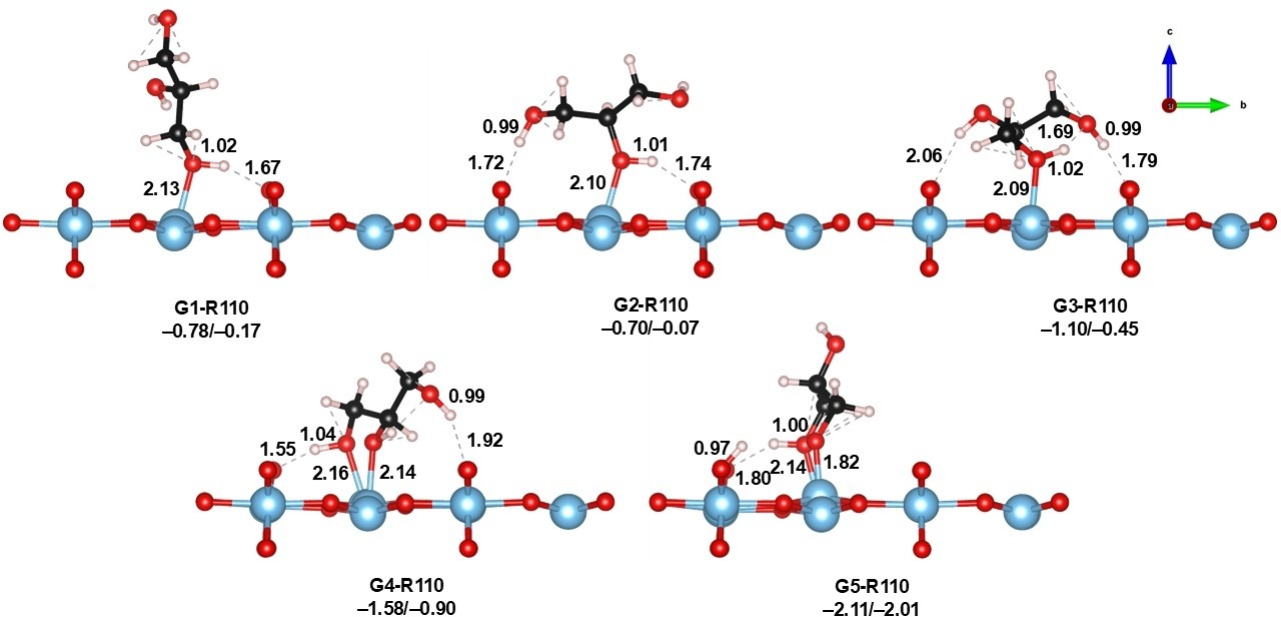
Optimized geometries of glycerol adsorbed on R110. Adsorption energies are also shown in terms of ΔE/ΔG_298_ (in eV). Distances are shown in Å.

The rutile crystalline phase of TiO_2_ has been widely studied due to its stability and ease of preparation. R110 has two kinds of Ti atoms, 5‐fold and 6‐fold‐coordinated (Ti_5c_ and Ti_6c_). Rows of Ti_6c_ long in the (001) direction alternate with rows of Ti_5c_ atoms that have one dangling bond perpendicular to the surface (see Figure 1c). Also, two‐fold (O_2c_) and 3‐fold‐cordinated (O_3c_) are also found on the surface.[^18^, ^19^] From all starting configurations, we successfully located five adsorption modes of glycerol on R110, from which only one is dissociative (see Figure 4).

The first mode, G1, shows the glycerol in a perpendicular configuration with respect to the surface, resembling the structure G1‐A101 (Figure 2). Here, an H‐bond interaction is formed with one of the O_2c_, at 1.67 Å, and the Ti_5c_‐O_gly_ is 2.13 Å. G2 is similar to G1 with the difference that the Ti_5c_−O_gly_ bond is formed across the central −OH of glycerol and the H‐bond between terminal −OH of glycerol and a vicinal bridged O_2c_ is somewhat larger (1.74 Å). G3 is formed via adsorption of the terminal −OH of glycerol, with a Ti_5c_−O_gly_ bond distance of 2.09 Å. In this mode, inter‐ and intramolecular networks of H−bond are formed, which helps to stabilize the glycerol. Finally, G4 and G5 correspond to bidentate states in which the terminal −OH's of glycerol interact with adjacent Ti_5c_ atoms. In G4, the Ti_5c_−O_gly_ bond distances are 2.14 and 2.16 Å, while in G5 one Ti_5c_−O_gly_ is slightly shorter at 1.82 Å. This is a consequence of the terminal glycerol −OH dissociation, leading to hydroxylation of the R110 surface without reconstruction. In terms of energies, all adsorptions are favorable, ranging from −2.11 to −0.70 eV. Adsorption modes G1 and G2 are very similar in energies (−0.78 *vs* −0.70 eV), suggesting no strong preference for any −OH of glycerol (terminal or central). However, G3 shows a more favorable adsorption energy compared to G1 (−1.10 eV), which could be due to the extended H−bond network between the glycerol and the surface. Thus, secondary interactions are important in this case. Comparison between G4 and G5 shows that the partially dissociative mode G5 is preferred over molecular mode G4, even though they both are bidentate configurations (−1.58 vs −2.11 eV). The strong adsorption in G5 correlates with the shorter Ti_5c_−O_gly_ bond distance of 1.82 Å compared to the Ti_5c_−O_gly_ distances in G4. The range of energies calculated for glycerol adsorption on the R110 surface suggests that its reactivity lies between that of A101 and A001, yet R110 does not undergo reconstruction. Few computational works have dealt with glycerol adsorption on R110.[^35^, ^70^] The calculated geometries and energies reported here are in reasonable agreement with the reported in the literature. Focusing on Gibbs energies, all adsorption modes on R110 are favorable, although G1 and G2 can be considered as weak modes due to the low value of adsorption energy.

### Role of Surface Defects on the Adsorption Energies

**Figure 5:**
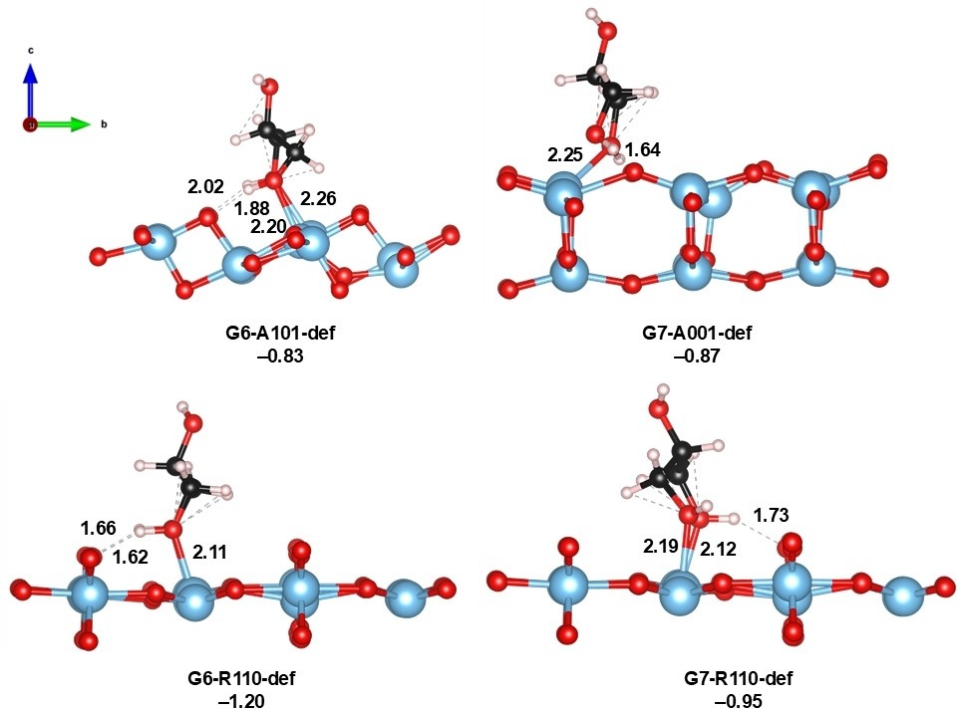
Optimized geometries of glycerol adsorbed on A101‐def, A001‐def and R110‐def surfaces with O vacancy defects. Adsorption energies are shown in terms of ΔE (in eV). Distances are shown in Å.

Surfaces with O vacancy defects (see Figure S3 of SI) were also probed during glycerol adsorption. For these surfaces, we focus on the most energetically favorable adsorption modes of the pristine surface, and the results can be seen in Figure 5. For the A101‐def surface, the adsorption energy of glycerol of the G6 mode is the same as in the G5 mode of the pristine surface (−0.83 eV), indicating that the presence of the O vacancy is irrelevant on this facet. This is due to the fact that the glycerol in this case does not adsorb directly on the O vacancy, but rather migrates during the geometry optimization process to the original position. Also, the local geometry of glycerol interacting with the surface is very similar to that of the mode G5 of the pristine surface, especially the Ti−O_gly_ and H_gly_⋅⋅⋅O bond distances. On the A001‐def surface, G7 is a molecular mode with the energy of −0,87 eV, much higher (i. e. less negative) than any energy reported in Figure 3 for the non‐defective surface. In this case, glycerol indeed absorbs directly on the O vacancy, interacting with the surrounding Ti atoms and exposed O atoms. Finally, for R110‐def, two adsorption modes G6 and G7 were localized, with energies of −1.20 and −0.95 eV, respectively. These two correspond to molecular modes where the terminal O_gly_ points towards the O_2c_ of the surface (in G6 the two OH points to one side while in G7 they point to the O vacancy). These two modes can be compared to modes G4 and G5 of the unmodified surface (Figure 4), which represents bidentate configurations. In this case, the adsorption energies are less favorable than those of the perfect surface, indicating that O vacancies indeed have an impact on the glycerol adsorption process. This agrees with the reactivity of R110 surfaces in other reactions both at experimental^[71]^ and computational levels,^[72]^ for instance, during the N_2_ reduction by water photolysis to form ammonia, where O vacancies have demonstrated a direct active role during the catalysis.

These results suggest that under more realistic conditions some differences are observed compared to the ideal pristine surfaces. Nevertheless, the calculations carried out here show that surfaces with O vacancy defects are also active for the adsorption of glycerol, given that all of them are favorable.

### Analysis of the Electronic Structure on Adsorbed States

**Figure 6:**
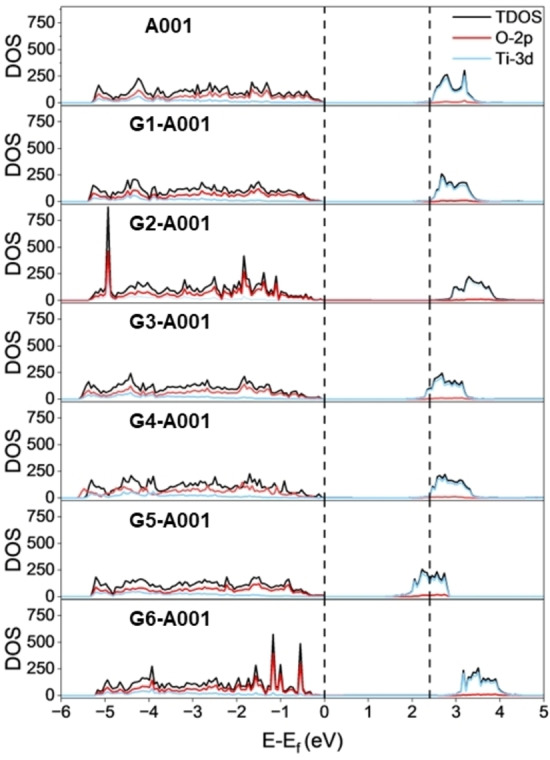
Total and Partial Density of States of adsorbed states G1 to G6 of the A001 surface. Only the states corresponding to the Ti 3d and O 2p of the surfaces are shown. The Fermi level is set at 0 eV.

We aim to analyze the electronic features of the glycerol adsorption on A101, A001, and R110. For this, we calculate the TDOS and PDOS of all adsorption modes of glycerol on TiO_2_. The results for the most reactive A001 surface are shown in Figure 6 and those for A101 and R110 are presented in the SI, Figure S4. In the bare surfaces, the valence states are clearly dominated by the O 2p orbitals, while Ti 3d orbitals largely contribute to the conduction band. The calculated band gap is about ~2.4 eV for anatase and ~1.8 eV for rutile, values that are clearly underestimated with respect to the experimental data. However, these values are in line with other works using GGA functionals, and the inclusion of hybrid functionals has proved to be necessary for the correct description of the band gap. Yet, hybrid functionals are prohibitively costly for the calculations carried out here. Nevertheless, as we are more interested in analyzing trends in the electronic structure of the TiO_2_ upon glycerol adsorption rather than reproducing an experimental value, we believe that the methodology used here is appropriate. For almost all adsorptions studied here, the main contributions to the valence and conduction bands of the TiO_2_ material are not affected by glycerol adsorption. It is worth mentioning, however, the main effect of the adsorbed state of glycerol on TiO_2_ is the modification of the band gap.

For the A101 and R110 surfaces all adsorptions lead to an increase in the band gap to values between 2.5 and 3 eV (for A101) and ~2.5 eV (for R110). This indicates that upon glycerol adsorption all surfaces become less conductive, which may have implications regarding photocatalytic reactions with alcohols, for instance. In contrast, the most reactive surface A001 shows both decrease (G3‐G5) and increase (G2, G6) of the band gap. Interestingly, the G1 mode, the only molecular mode with no surface reconstruction, shows virtual no change in the band gap compared to the bare A001 surface. Following these trends, we observed that H‐bonding between glycerol and the surface, surface modification, and the mode glycerol adsorbs on A001 are important to describe the electronic changes in TiO_2_ upon glycerol adsorption. We note that modes G3‐G5 in A001 show a decrease in the band gap compared to bare A001, although the decrease in G3 and G4 is modest compared to G5. A common feature of these three adsorption states is that glycerol interacts with the surface through the central −OH group, which leaves one terminal −OH group available to form H‐bonding with one of the O_2c_ of the surface. Since G3 and G4 adsorbed states are similar in geometry and energy (see above), the effect on the band gap is similar in both states. In G5 there is no proton transfer to the surface, yet the surface undergoes modification. This is equivalent to creating a defect on the surface that ultimately decreases the band gap. On the other hand, modes G2 and G6 feature glycerol adsorption through the terminal −OH. This prevents the formation of additional stabilizing H‐bonding between the other −OH groups and the surface. This effect suggests that the main parameters for defining the electronic structure of the TiO_2_ upon glycerol adsorption are the coordination mode (central *vs*. terminal −OH) and the formation of H‐bonding.

**Figure 7:**
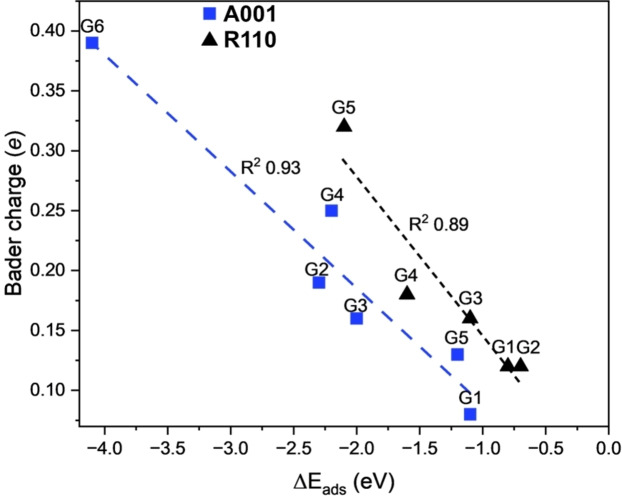
Relationship between the Bader charge of glycerol adsorbed on TiO_2_ (charge transfer) and the adsorption energies for A001 (blue) and R110 (black). The R^2^ value of the linear regression is shown.

Finally, we also compute Charge Density Difference (CDD) maps and Bader charges analysis to further understand the glycerol adsorption process. Figure S5 of the SI shows the CDD maps for selected adsorption modes on the three surfaces. As observed, the adsorption of glycerol is accompanied by charge redistribution at the surfaces. In general, we see zones of charge accumulation around the C−O bond of glycerol and charge depletion on the C−H bonds. The Bader charge analysis (see Table S1) shows that the overall charge on the glycerol molecule adsorbed on TiO_2_ is positive (calculated as the summation over all Bader atomic charges of the glycerol adsorbed on the surface), suggesting charge donation from glycerol to the surface. For the A001 and R110 surfaces, we observe that the more favorable adsorption modes are associated with large charge transfer glycerol→TiO_2_. This is evident from Figure 7, which shows excellent linear correlations between the amount of charge transfer to the surface and the adsorption energies. These two surfaces are the most reactive surfaces of the three studied systems. However, for the A101 surface, we did not find any correlation between charge transfer and the adsorption energies, possibly due to the small values of adsorption energies (see Figure S6 in SI). Nevertheless, we can establish that the charge transfer can be used as a molecular descriptor for understanding the adsorption process on A001 and R110. In this case, the more favorable adsorption processes (bidentate and/or dissociative) are associated with a large charge transfer process, while the less favorable molecular modes involve weak charge transfer.

## Conclusions

In this work, we carried out a systematic study of glycerol adsorption on anatase (101), anatase (001), and rutile (110) TiO_2_ oxides using computational chemistry based on DFT. We explored several adsorption modes on these surfaces with starting configuration without bias. Of the three studied surfaces, anatase (101) is found to be the less reactive one, with adsorption energies between −0.8 and −0.4 eV. On this surface, all glycerol adsorption modes are molecular. Anatase (001) is the most reactive surface, and in this surface both molecular and dissociative adsorption modes, few of them featuring double dissociated states, with energies ranging from −4 to −1 eV. Here, severe surface reconstructions are observed in some cases. Rutile (110) also shows both molecular and dissociative adsorptions, but it is less reactive than anatase (001). The inclusion of enthalpy and entropy contributions to the electronic energies does not change the nature of most of the adsorption states, except for G1 and G2 of A101, which become slightly unfavorable. Moreover, we also consider some cases of glycerol adsorption on surfaces with O vacancy defects, which are the most common point defects in TiO_2_. In this case, the geometric configuration and energies are different from those of the pristine surfaces, except again for A101, for which the adsorption energy is the same as that of the unmodified surface. We also characterized the electronic structure of the TiO_2_ surfaces before and after glycerol adsorption. The TDOS and PDOS analysis revealed that glycerol adsorption mainly affects the band gap of the TiO_2_ and not the individual contributions to the valence and conduction band. Bader charge analysis shows that strong adsorption modes on anatase (001) and rutile (110) are associated with large charge transfer from glycerol to the surface, while weak and molecular adsorption modes involve low char transfer. Thus, we propose that the Bader charges can be used as a molecular descriptor to understand glycerol adsorption processes on TiO_2_.

## Conflict of Interests

The authors declare no conflict of interest.

1

## Supporting information

As a service to our authors and readers, this journal provides supporting information supplied by the authors. Such materials are peer reviewed and may be re‐organized for online delivery, but are not copy‐edited or typeset. Technical support issues arising from supporting information (other than missing files) should be addressed to the authors.

Supporting Information

## Data Availability

The data that support the findings of this study are available from the corresponding author upon reasonable request.

## References

[1] M. Braun , C. S. Santana , A. C. Garcia , C. Andronescu , Curr. Opin. Green Sustain. Chem. 2023, 41, 100829.

[2] K. Mohan , S. D. K. R. Pai , B. S. Reghunath , D. Pinheiro , ChemistrySelect 2023, 8, e202204501.

[3] J. Charles Rajesh Kumar , M. A. Majid , Energy. Sustain. Soc. 2020, 10, Doi: 10.1186/s13705-019-0232-1.

[4] C. Decarpigny , A. Aljawish , C. His , B. Fertin , M. Bigan , P. Dhulster , M. Millares , R. Froidevaux , Energies 2022, 15, Doi: 10.3390/en15093381.

[5] L. T. Thanh , K. Okitsu , L. Van Boi , Y. Maeda , Catalysts 2012, 2, 191–222.

[6] H. H. Kuo , T. G. Vo , Y. J. Hsu , J. Photochem. Photobiol. C Photochem. Rev. 2024, 58, 100649.

[7] Z. Pirzadi , F. Meshkani , Fuel 2022, 329, 125044.

[8] M. Anitha , S. K. Kamarudin , N. T. Kofli , Chem. Eng. J. 2016, 295, 119–130.

[9] M. Checa , S. Nogales-Delgado , V. Montes , J. M. Encinar , Catalysts 2020, 10, 1–41.

[10] Y. Liu , B. Zhang , D. Yan , X. Xiang , Green Chem. 2024, 26, 2505–2524.

[11] G. Dodekatos , S. Schünemann , H. Tüysüz , ACS Catal. 2018, 8, 6301–6333.

[12] S. Torres , R. Palacio , D. López , Appl. Catal. A Gen. 2021, 621, 118199.

[13] Y. C. Lin , Int. J. Hydrogen Energy 2013, 38, 2678–2700.

[14] M. H. M. Pires , F. B. Passos , Y. Xing , Catal. Today 2023, 419, 114161.

[15] X. Han , H. Sheng , C. Yu , T. W. Walker , G. W. Huber , J. Qiu , S. Jin , ACS Catal. 2020, 10, 6741–6752.

[16] P. D. Vaidya , A. E. Rodrigues , Chem. Eng. Technol. 2009, 32, 1463–1469.

[17] P. Anastas , N. Eghbali , Chem. Soc. Rev. 2010, 39, 301–312. 20023854 10.1039/b918763b

[18] K. Bourikas , C. Kordulis , A. Lycourghiotis , Chem. Rev. 2014, 114, 9754–9823. 25253646 10.1021/cr300230q

[19] U. Diebold , Surf. Sci. Rep. 2003, 48, 53–229.

[20] M. G. Rinaudo , A. M. Beltrán , A. Fernández , L. E. Cadús , M. R. Morales , Results Eng. 2022, 16, 100737.

[21] F. J. López-Tenllado , J. Hidalgo-Carrillo , V. Montes , A. Marinas , F. J. Urbano , J. M. Marinas , L. Ilieva , T. Tabakova , F. Reid , Catal. Today 2017, 280, 58–64.

[22] G. Wu , Y. Liu , Y. He , J. Feng , D. Li , Appl. Catal. B Environ. 2021, 291, 120061.

[23] P. Panagiotopoulou , E. E. Karamerou , D. I. Kondarides , Catal. Today 2013, 209, 91–98.

[24] X. Zhang , M. Gao , P. Yang , X. Cui , Y. Liu , D. Li , J. Feng , J. Porous Mater. 2021, 28, 1371–1385.

[25] C. D'Agostino , G. Brett , G. Divitini , C. Ducati , G. J. Hutchings , M. D. Mantle , L. F. Gladden , ACS Catal. 2017, 7, 4235–4241.

[26] W. Mondach , S. Chanklang , P. Somchuea , T. Witoon , M. Chareonpanich , K. Faungnawakij , H. Sohn , A. Seubsai , Sci. Rep. 2021, 11, 23042. 34845268 10.1038/s41598-021-02416-7 PMC8630069

[27] N. Rojas , G. Hincapié-Triviño , M. Velasquez , Mol. Catal. 2024, 566, 114390.

[28] V. Augugliaro , H. A. H. El Nazer , V. Loddo , A. Mele , G. Palmisano , L. Palmisano , S. Yurdakal , Catal. Today 2010, 151, 21–28.

[29] T. Jedsukontorn , V. Meeyoo , N. Saito , M. Hunsom , Chem. Eng. J. 2015, 281, 252–264.

[30] P. Huo , P. Kumar , B. Liu , Catalysts 2018, 8, 616.

[31] Q. Guo , Z. Ma , C. Zhou , Z. Ren , X. Yang , Chem. Rev. 2019, 119, 11020–11041. 31503466 10.1021/acs.chemrev.9b00226

[32] C. T. Campbell , J. R. V. Sellers , Chem. Rev. 2013, 113, 4106–4135. 23441680 10.1021/cr300329s

[33] R. Wang , B. Wang , A. S. Abdullahi , H. Fan , Wiley Interdiscip. Rev. Comput. Mol. Sci. 2024, 14, Doi: 10.1002/wcms.1686.

[34] Z. Dohnálek , I. Lyubinetsky , R. Rousseau , Prog. Surf. Sci. 2010, 85, 161–205.

[35] G. Carchini , N. López , Phys. Chem. Chem. Phys. 2014, 16, 14750–14760. 24919422 10.1039/c4cp01546k

[36] Z. Babaei , A. Najafi Chermahini , M. Dinari , J. Colloid Interface Sci. 2020, 563, 1–7. 31865043 10.1016/j.jcis.2019.12.051

[37] F. Jia , H. Zhou , M. Wang , Chem. Commun. 2023, 59, 11377–11380. 10.1039/d3cc03803c 37665623

[38] M. Ernzerhof , G. E. Scuseria , J. Chem. Phys. 1999, 110, 5029–5036.

[39] G. Kresse , J. Hafner , Phys. Rev. B 1993, 47, 558–561. 10.1103/physrevb.47.558 10004490

[40] G. Kresse , J. Hafner , Phys. Rev. B 1994, 49, 14251–14269. 10.1103/physrevb.49.14251 10010505

[41] G. Kresse , J. Furthmüller , Phys. Rev. B 1996, 54, 11169–11186. 10.1103/physrevb.54.11169 9984901

[42] G. Kresse , J. Hafner , J. Phys. Condens. Matter 1994, 6, 8245–8257.

[43] D. Joubert , Phys. Rev. B - Condens. Matter Mater. Phys. 1999, 59, 1758–1775.

[44] S. Grimme , J. Antony , S. Ehrlich , H. Krieg , J. Chem. Phys. 2010, 132, 154104. 20423165 10.1063/1.3382344

[45] A. Jain , S. P. Ong , G. Hautier , W. Chen , W. D. Richards , S. Dacek , S. Cholia , D. Gunter , D. Skinner , G. Ceder , K. A. Persson , APL Mater. 2013, 1, 011002.

[46] K. Momma , F. Izumi , J. Appl. Crystallogr. 2008, 41, 653–658.

[47] M. K. Nowotny , L. R. Sheppard , T. Bak , J. Nowotny , J. Phys. Chem. C 2008, 112, 5275–5300.

[48] H. Zhao , F. Pan , Y. Li , J. Mater. 2017, 3, 17–32.

[49] X. Pan , M. Q. Yang , X. Fu , N. Zhang , Y. J. Xu , Nanoscale 2013, 5, 3601–3614. 23532413 10.1039/c3nr00476g

[50] Y. Ortega , D. F. Hevia , J. Oviedo , M. A. San-Miguel , Appl. Surf. Sci. 2014, 294, 42–48.

[51] Y. Shi , H. Sun , M. C. Nguyen , C. Wang , K. Ho , W. A. Saidi , J. Zhao , Nanoscale 2017, 9, 11553–11565. 28770922 10.1039/c7nr02458d

[52] Y. Han , C. J. Liu , Q. Ge , J. Phys. Chem. C 2007, 111, 16397–16404.

[53] L. Kou , T. Frauenheim , A. L. Rosa , E. N. Lima , J. Phys. Chem. C 2017, 121, 17417–17420.

[54] B. M. Comer , M. H. Lenk , A. P. Rajanala , E. L. Flynn , A. J. Medford , Catal. Letters 2021, 151, 1142–1154.

[55] V. Wang , N. Xu , J. C. Liu , G. Tang , W. T. Geng , Comput. Phys. Commun. 2021, 267, 108033.

[56] G. Henkelman , A. Arnaldsson , H. Jónsson , Comput. Mater. Sci. 2006, 36, 354–360.

[57] E. Sanville , S. D. Kenny , R. Smith , G. Henkelman , J. Comput. Chem. 2007, 28, 899–908. 17238168 10.1002/jcc.20575

[58] W. Tang , E. Sanville , G. Henkelman , J. Phys. Condens. Matter 2009, 21, 084204. 21817356 10.1088/0953-8984/21/8/084204

[59] M. Yu , D. R. Trinkle , J. Chem. Phys. 2011, 134, 064111. 21322665 10.1063/1.3553716

[60] C. R. O'Connor , R. Ma , G. Collinge , M. S. Lee , G. A. Kimmel , Z. Dohnálek , Top. Catal. 2023, 66, 1087–1101.

[61] A. Tilocca , A. Selloni , J. Phys. Chem. B 2004, 108, 19314–19319.

[62] M. Setvin , X. Shi , J. Hulva , T. Simschitz , G. S. Parkinson , M. Schmid , C. Di Valentin , A. Selloni , U. Diebold , ACS Catal. 2017, 7, 7081–7091. 29034122 10.1021/acscatal.7b02003 PMC5634753

[63] F. H. Tian , X. Wang , W. Zhao , L. Zhao , T. Chu , S. Yu , Surf. Sci. 2013, 616, 76–84.

[64] Y. Wu , F. Gao , H. Wang , L. Kovarik , B. Sudduth , Y. Wang , J. Phys. Chem. C 2021, 125, 3988–4000.

[65] M. Yao , Y. Ji , H. Wang , Z. Ao , G. Li , T. An , J. Phys. Chem. C 2017, 121, 13717–13722.

[66] L. A. A. Varilla , N. Seriani , J. A. Montoya , J. Mol. Model. 2019, 25, 231. 31324989 10.1007/s00894-019-4103-7

[67] L. Liu , Z. Wang , C. Pan , W. Xiao , K. Cho , ChemPhysChem 2013, 14, 996–1002. 23460451 10.1002/cphc.201201048

[68] S. Kenmoe , O. Lisovski , S. Piskunov , D. Bocharov , Y. F. Zhukovskii , E. Spohr , J. Phys. Chem. B 2018, 122, 5432–5440. 29596747 10.1021/acs.jpcb.7b11697

[69] X. Q. Gong , A. Selloni , J. Phys. Chem. B 2005, 109, 19560–19562. 16853530 10.1021/jp055311g

[70] C. Rohmann , H. Idriss , J. Phys. Condens. Matter 2022, 34, 154002. 10.1088/1361-648X/ac4d5b 35051917

[71] H. Hirakawa , M. Hashimoto , Y. Shiraishi , T. Hirai , J. Am. Chem. Soc. 2017, 139, 10929–10936. 28712297 10.1021/jacs.7b06634

[72] X. Y. Xie , P. Xiao , W. H. Fang , G. Cui , W. Thiel , ACS Catal. 2019, 9, 9178–9187.

