# Supplementary material for: Glycerol Adsorption on TiO2 Surfaces: A Systematic Periodic DFT Study
Source: ChemistryOpen. 2025 Jan 28;14(4):e202400153. doi: 10.1002/open.202400153 (PMC11973501; doi:10.1002/open.202400153)
Supplement: Supplementary file 1 — Supporting Information [file OPEN-14-e202400153-s001.pdf]

ChemistryOpen

Supporting Information

Glycerol Adsorption on TiO_2 Surfaces: A Systematic Periodic DFT Study

Andrés Camilo Muñoz Peña, Elizabeth Flórez, and Francisco Núñez-Zarur*

Supporting Information

Glycerol Adsorption on TiO_2 Surfaces: A Systematic Periodic DFT Study

Andrés Camilo Muñoz Peña,[1,2] Elizabeth Flórez,[2] Francisco Núñez-Zarur,[2,3*]

[1]Chemistry and Biochemistry Department, New Mexico State University, 88001, Las Cruces, NM, USA

[2]Facultad de Ciencias Básicas, Universidad de Medellín, 050026 Medellín, Colombia

[3]Departamento de Química, Facultad de Ciencias, Universidad Nacional de Colombia − Sede Bogotá, Carrera 30 No., 45-03, 111321 Bogotá, Colombia

Contents

Figure S1. Unit cell relaxation at several *k*-point grids for anatase and rutile. S2

Figure S2. Optimized surface structures of A101-def, A001-def, andR110-def with O vacancy defects. S3

Figure S3. Glycerol structures used in this study. S4

Figure S4. Total and Partial Density of States of adsorbed states G1 to G5 of the A101 and R110 surfaces. S5

Figure S5. Charge Density Difference (CDD) maps for selected adsorption modes of glycerol on A101, A001, and R100. S6

Table S1. Charge transfer (*e*) for the adsorption of glycerol on A101, A001, and R110. S7

Figure S6. Relationship between the Bader charge of glycerol adsorbed on TiO_2 (charge transfer) and the adsorption energies for A101. S8

Cartesian Coordinates of Bare Surface and Glycerol Adsorption Structures S9

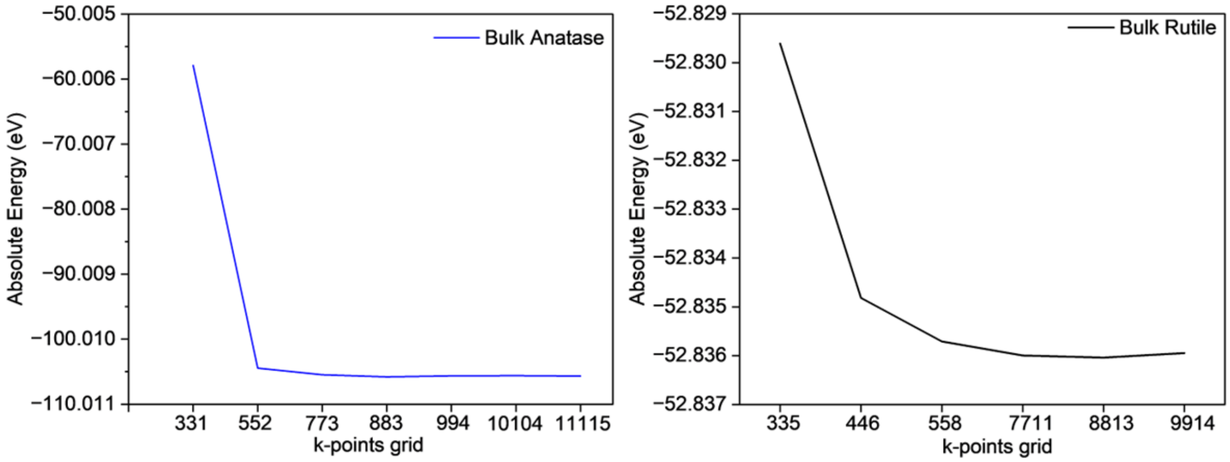

Figure S1. Unit cell relaxation at several *k*-point grids for anatase and rutile respectively.

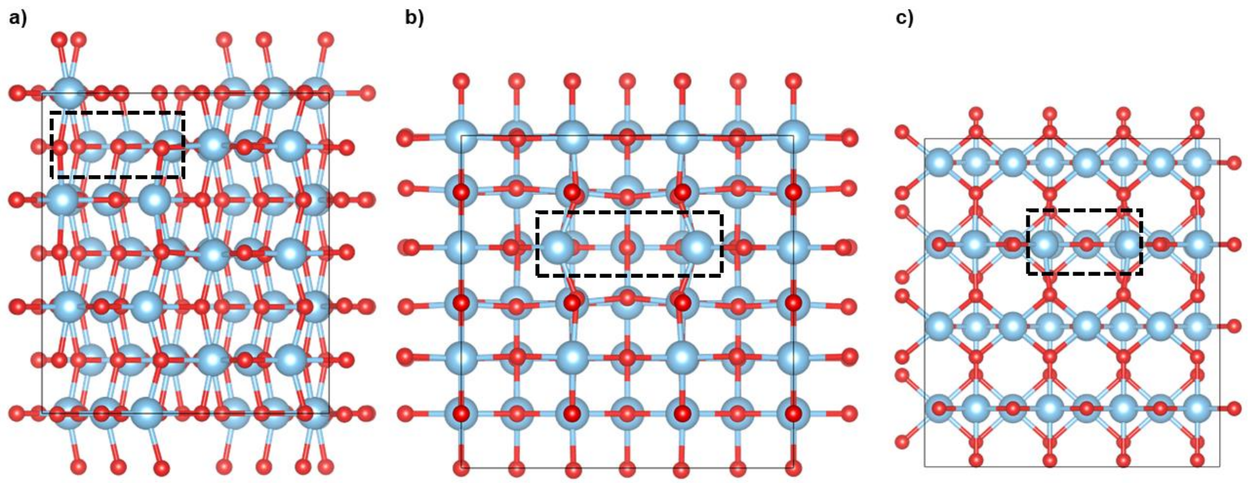

Figure S2. Optimized surface structures of a) A101-def; b) A001-def; c) R110-def with O vacancy defects. The exact position of the O vacant site is highlighted with the black dashed square.

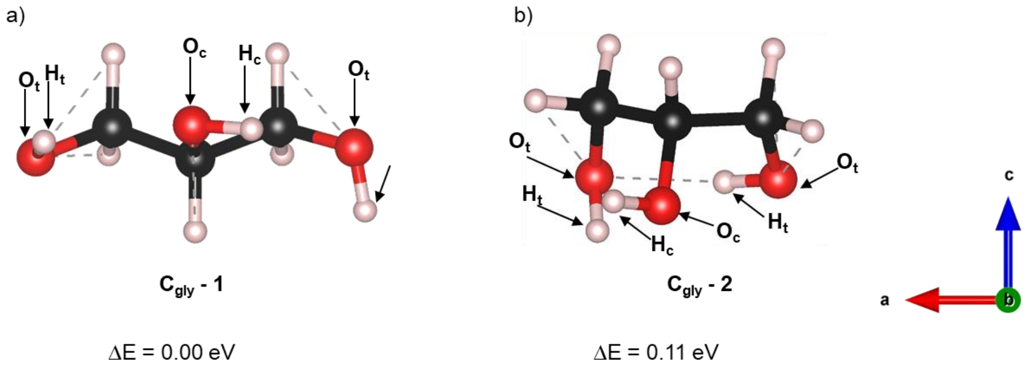

Figure S3. Glycerol structures used in this study. The C_{gly-1} is the most stable conformer while the C_{gly-2} lies only 0.11 eV above in energy. The latter isomer was used in glycerol adsorption process for bidentate states.

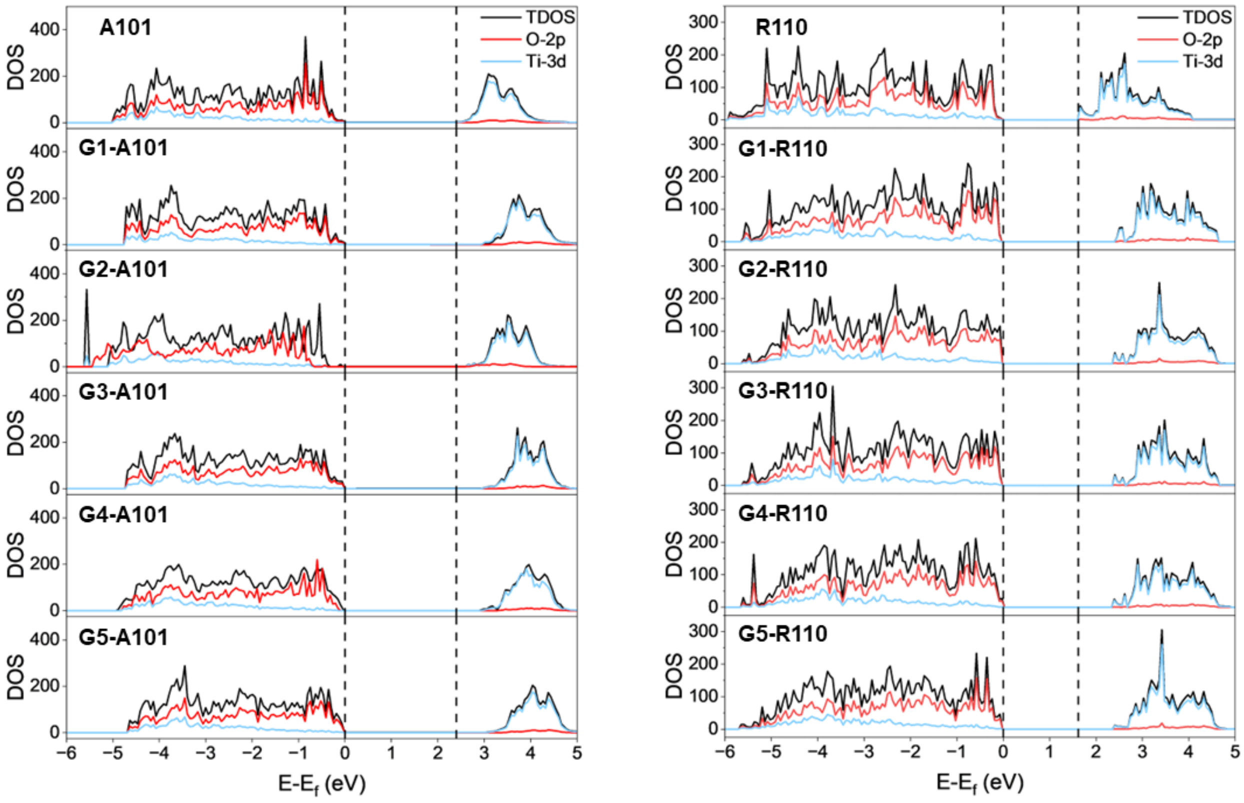

Figure S4. Total and Partial Density of States of adsorbed states G1 to G5 of the A101 and R110 surfaces. Only the states corresponding to the Ti 3d and O 2p of the surfaces are shown. The Fermi level is set at 0 eV.

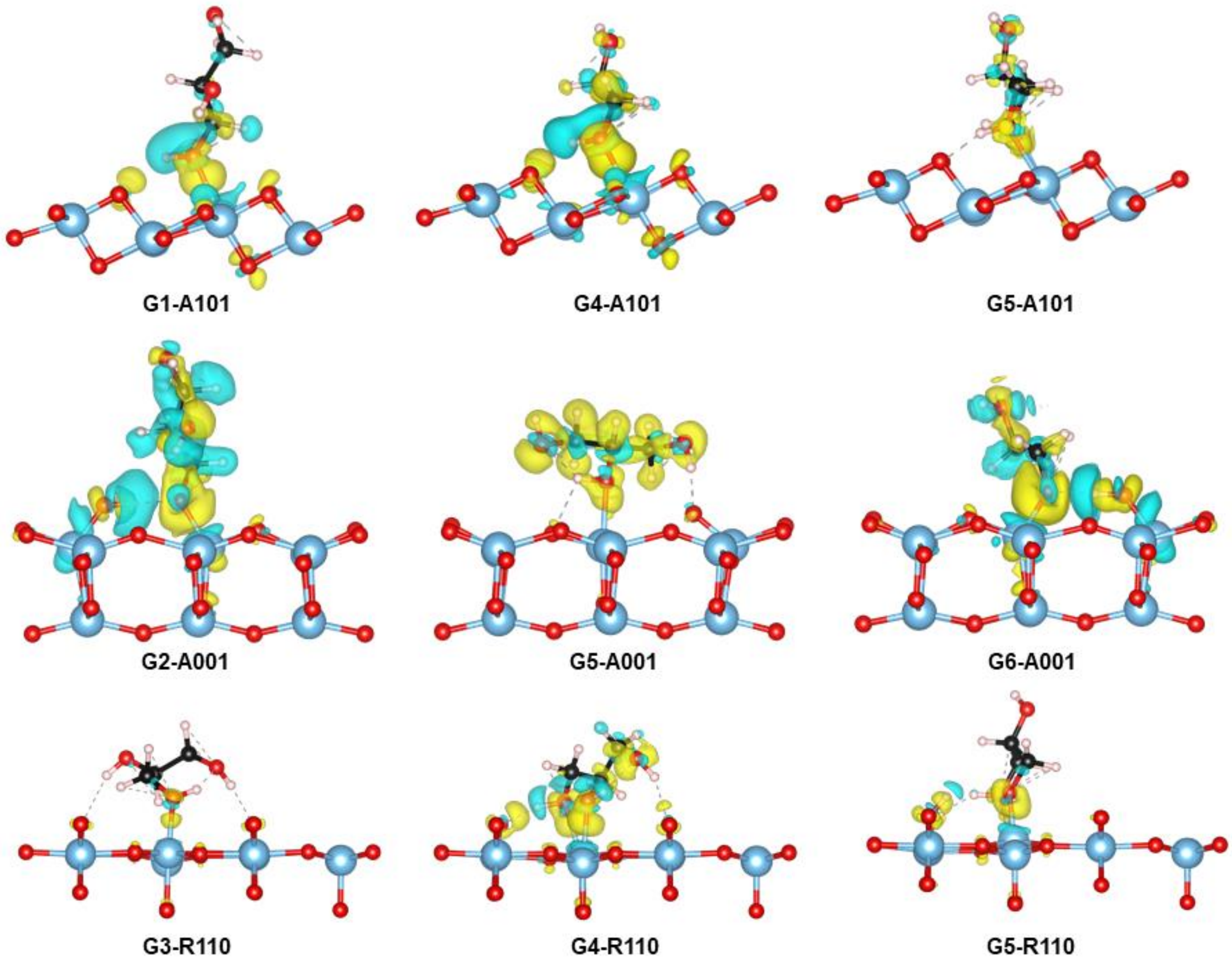

Figure S5. Charge Density Difference (CDD) maps for selected adsorption modes of glycerol adsorbed on A101 (up), A001 (middle), and R100 (bottom). Zones of charge accumulation are shown in yellow and charge depletion are shown in cyan. Isosurface value: 0.005 e/Å^3.

Table S1. Charge transfer (*e*) for the adsorption of glycerol on A101, A001, and R110. These values are calculated as the summation over all Bader atomic charges of the glycerol adsorbed on TiO_2.

	**A101**	**A001**	**R110**
**G1**	0.08	0.08	0.12
**G2**	0.12	0.19	0.12
**G3**	0.08	0.16	0.16
**G4**	0.13	0.25	0.18
**G5**	0.12	0.13	0.32
**G6**		0.39	

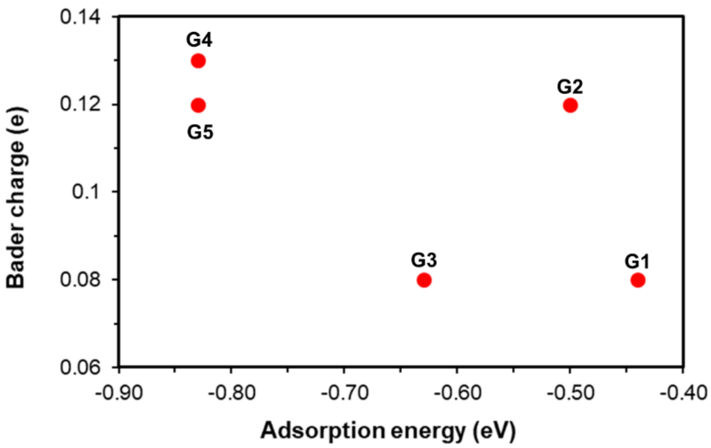

Figure S6. Relationship between the Bader charge of glycerol adsorbed on TiO_2 (charge transfer) and the adsorption energies for A101. No linear correlation for this surface was observed.

Cartesian Coordinates of Bare Surface and Glycerol Adsorption Structures

CONTCAR A101

H 8 C 3 O 99 Ti 48
1.0

10.2208995819	0.0000000000	0.0000000000
0.0000000000	11.4083003998	0.0000000000
0.0000000000	0.0000000000	32.9515991211

H	C	O	Ti
8	3	99	48

G1

5.159056664	4.756958485	18.010496140
5.302128792	4.255431652	15.336643219
3.536578178	3.318014860	19.235506058
3.959900379	2.995784760	16.669015884
3.559990406	6.049777508	19.973865509
4.180476665	6.574539185	16.851934433
2.217480421	4.455447197	18.823122025
2.636983633	4.162668228	16.338171005
4.074562550	4.856455803	17.804672241
3.301851988	4.372333527	19.030044556
3.706879377	4.061454773	16.559080124
4.737797260	0.855622470	2.479939699
6.152677536	0.855622470	6.009709835
7.559561253	0.858331621	9.602225304
9.063879013	0.868869007	13.453800201
6.228307724	2.757042646	1.882519841
7.643187523	2.757042646	5.412300110
9.042474747	2.762075424	8.971817970
0.272054195	2.765280485	12.598834991
8.429887772	0.855622470	1.000079989
9.844667435	0.855622470	4.529860020
1.029093266	0.867704570	8.085964203
2.503465176	0.814280748	11.642596245
2.536317587	2.757042646	3.362380028
3.951197147	2.757042646	6.892159939
5.387453079	2.761865616	10.501958847
6.893179893	2.814166784	14.063230515
9.848246574	2.757042646	2.479939699
1.042227387	2.757042646	6.009709835
2.455885649	2.759732485	9.634305954
3.942096949	2.728754044	13.367686272
1.117857456	0.855622470	1.882519841
2.532737255	0.855622470	5.412300110
3.928691626	0.860457778	8.975695610
5.406488419	0.852665126	12.584505081
3.319437265	2.757042646	1.000079989
4.734217644	2.757042646	4.529860020
6.138489723	2.760828972	8.086810112
7.626903057	2.749866724	11.646516800

7.646767139	0.855622470	3.362380028
9.061646461	0.855622470	6.892159939
0.266717613	0.864662588	10.505364418
1.823327661	0.874217689	14.064505577
4.737797260	4.658352375	2.479939699
6.152677536	4.658352375	6.009709835
7.566638947	4.660871506	9.609111786
9.061295509	4.665084362	13.446020126
6.228307724	6.559772491	1.882519841
7.643187523	6.559772491	5.412300110
9.044996262	6.559371471	8.973357201
0.282594413	6.564136982	12.597328186
8.429887772	4.658352375	1.000079989
9.844667435	4.658352375	4.529860020
1.041305423	4.658339977	8.104122162
2.517844915	4.664474487	11.714191437
2.536317587	6.559772491	3.362380028
3.951197147	6.559772491	6.892159939
5.386489868	6.562088490	10.501029015
6.913534164	6.547122002	14.060091972
9.848246574	6.559772491	2.479939699
1.042227387	6.559772491	6.009709835
2.454902649	6.561126232	9.633263588
3.947080851	6.595753670	13.395407677
1.117857456	4.658352375	1.882519841
2.532737255	4.658352375	5.412300110
3.928058863	4.660006523	8.983923912
5.373190403	4.665600300	12.588371277
3.319437265	6.559772491	1.000079989
4.734217644	6.559772491	4.529860020
6.139256477	6.559282780	8.086035728
7.626810551	6.567423344	11.645298004
7.646767139	4.658352375	3.362380028
9.061646461	4.658352375	6.892159939
0.270582616	4.660277843	10.519109726
1.784148097	4.658897877	14.114373207
4.737797260	8.461192131	2.479939699
6.152677536	8.461192131	6.009709835
7.561253548	8.462625504	9.603790283
9.069260597	8.464217186	13.444149017
6.228307724	10.362502098	1.882519841
7.643187523	10.362502098	5.412300110
9.043883324	10.364586830	8.975898743
0.280003071	10.372072220	12.600376129
8.429887772	8.461192131	1.000079989
9.844667435	8.461192131	4.529860020
1.030489445	8.453595161	8.085843086
2.509031057	8.504480362	11.643491745
2.536317587	10.362502098	3.362380028
3.951197147	10.362502098	6.892159939
5.384572983	10.366930008	10.502909660

6.936611176	10.365907669	14.078343391
9.848246574	10.362502098	2.479939699
1.042227387	10.362502098	6.009709835
2.443372488	10.365367889	9.584537506
3.978462458	10.366560936	13.466122627
1.117857456	8.461192131	1.882519841
2.532737255	8.461192131	5.412300110
3.928614855	8.461587906	8.974301338
5.403025150	8.472583771	12.579089165
3.319437265	10.362502098	1.000079989
4.734217644	10.362502098	4.529860020
6.142062187	10.363146782	8.086138725
7.632228374	10.368876457	11.655556679
7.646767139	8.461192131	3.362380028
9.061646461	8.461192131	6.892159939
0.269344330	8.463930130	10.505247116
1.821933270	8.465616226	14.063483238
3.658324480	5.105298042	20.197296143
4.388828754	4.607657909	15.389744759
3.733070135	6.240643501	17.650182724
6.583897591	0.855622470	1.739840031
7.998667717	0.855622470	5.269619942
9.403237343	0.856679440	8.869064331
0.659323215	0.869681418	12.590001106
4.382317543	2.757042646	2.622289896
5.797186852	2.757042646	6.152059555
7.154922485	2.766123533	9.686038017
8.536396980	2.771816969	13.209931374
1.473447323	2.757042646	1.739840031
2.888217449	2.757042646	5.269619942
4.288733482	2.762502909	8.862739563
5.751088619	2.763379097	12.534533501
9.492767334	0.855622470	2.622289896
0.686737418	0.855622470	6.152059555
2.028158665	0.857396543	9.693735123
3.448197603	0.840813518	13.183954239
6.583897591	4.658352375	1.739840031
7.998667717	4.658352375	5.269619942
9.431488991	4.662280083	8.862772942
0.683158219	4.663249016	12.592590332
4.382317543	6.559772491	2.622289896
5.797186852	6.559772491	6.152059555
7.149487972	6.556021690	9.689041138
8.549774170	6.559550285	13.202195168
1.473447323	6.559772491	1.739840031
2.888217449	6.559772491	5.269619942
4.291244984	6.559826374	8.867515564
5.763704300	6.564620018	12.559434891
9.492767334	4.658352375	2.622289896
0.686737418	4.658352375	6.152059555
2.041490078	4.660527229	9.757268906

3.458242893	4.670797348	13.333569527
6.583897591	8.461192131	1.739840031
7.998667717	8.461192131	5.269619942
9.405138016	8.463264465	8.868772507
0.662731946	8.464519501	12.587907791
4.382317543	10.362502098	2.622289896
5.797186852	10.362502098	6.152059555
7.141629696	10.363998413	9.695735931
8.566233635	10.369470596	13.207951546
1.473447323	10.362502098	1.739840031
2.888217449	10.362502098	5.269619942
4.294609070	10.365509033	8.868391991
5.792379379	10.365931511	12.592807770
9.492767334	8.461192131	2.622289896
0.686737418	8.461192131	6.152059555
2.030529737	8.463652611	9.694067001
3.448957443	8.482785225	13.188801765

G2

3.333718300	4.528817177	18.376911163
3.599475145	6.860018253	17.499612808
2.588516951	4.016417027	16.834550858
2.898827076	6.450700760	15.913783073
4.756130219	2.788388014	18.163612366
5.426877975	5.165910244	17.082126617
5.418746948	7.029459000	15.376619339
5.276965618	3.847726583	15.268301010
3.542814493	4.133826256	17.369052887
3.840861797	6.506969452	16.484895706
4.436102390	5.109523296	16.597112656
4.737797260	0.855622470	2.479939699
6.152677536	0.855622470	6.009709835
7.566065788	0.851855457	9.596423149
9.062520981	0.857881069	13.449286461
6.228307724	2.757042646	1.882519841
7.643187523	2.757042646	5.412300110
9.053309441	2.757228613	8.970160484
0.275031358	2.761159658	12.615224838
8.429887772	0.855622470	1.000079989
9.844667435	0.855622470	4.529860020
1.034847379	0.853308558	8.088938713
2.511156321	0.815218031	11.653463364
2.536317587	2.757042646	3.362380028
3.951197147	2.757042646	6.892159939
5.394686699	2.740677834	10.485132217
6.890615940	2.798613787	14.050611496
9.848246574	2.757042646	2.479939699
1.042227387	2.757042646	6.009709835
2.456661463	2.751356840	9.624784470
3.970817804	2.714863300	13.387246132
1.117857456	0.855622470	1.882519841

2.532737255	0.855622470	5.412300110
3.931500435	0.852511168	8.975620270
5.404451847	0.834148228	12.563411713
3.319437265	2.757042646	1.000079989
4.734217644	2.757042646	4.529860020
6.142219067	2.760623217	8.080111504
7.639632702	2.738898516	11.635406494
7.646767139	0.855622470	3.362380028
9.061646461	0.855622470	6.892159939
0.272122115	0.858249426	10.511902809
1.824282646	0.867665410	14.071502686
4.737797260	4.658352375	2.479939699
6.152677536	4.658352375	6.009709835
7.572082520	4.655949116	9.601214409
9.058835030	4.658906937	13.455425262
6.228307724	6.559772491	1.882519841
7.643187523	6.559772491	5.412300110
9.054684639	6.557681561	8.966818810
0.281035990	6.552406788	12.606769562
8.429887772	4.658352375	1.000079989
9.844667435	4.658352375	4.529860020
1.046677470	4.657151222	8.105634689
2.512297392	4.608815193	11.713382721
2.536317587	6.559772491	3.362380028
3.951197147	6.559772491	6.892159939
5.396670341	6.572544098	10.483000755
6.885047913	6.548847198	14.036347389
9.848246574	6.559772491	2.479939699
1.042227387	6.559772491	6.009709835
2.462503672	6.557183743	9.656242371
3.932711124	6.548401833	13.297767639
1.117857456	4.658352375	1.882519841
2.532737255	4.658352375	5.412300110
3.931931019	4.657002926	8.987172127
5.383262157	4.638551712	12.508051872
3.319437265	6.559772491	1.000079989
4.734217644	6.559772491	4.529860020
6.139655590	6.551836014	8.079560280
7.638843536	6.554755688	11.626886368
7.646767139	4.658352375	3.362380028
9.061646461	4.658352375	6.892159939
0.270908445	4.660778999	10.521723747
1.808445454	4.669902802	14.123444557
4.737797260	8.461192131	2.479939699
6.152677536	8.461192131	6.009709835
7.569337845	8.458634377	9.593929291
9.069766045	8.449067116	13.457428932
6.228307724	10.362502098	1.882519841
7.643187523	10.362502098	5.412300110
9.056886673	10.359633446	8.980338097
0.291054547	10.357937813	12.627780914

8.429887772	8.461192131	1.000079989
9.844667435	8.461192131	4.529860020
1.041341901	8.453581810	8.098099709
2.520412207	8.510740280	11.692494392
2.536317587	10.362502098	3.362380028
3.951197147	10.362502098	6.892159939
5.391608238	10.348694801	10.499130249
6.939892292	10.355744362	14.078614235
9.848246574	10.362502098	2.479939699
1.042227387	10.362502098	6.009709835
2.453155041	10.358717918	9.606896400
3.980601788	10.357642174	13.475889206
1.117857456	8.461192131	1.882519841
2.532737255	8.461192131	5.412300110
3.926832438	8.454985619	8.975486755
5.397397041	8.456014633	12.583047867
3.319437265	10.362502098	1.000079989
4.734217644	10.362502098	4.529860020
6.145852566	10.360723495	8.085539818
7.636322021	10.349937439	11.653856277
7.646767139	8.461192131	3.362380028
9.061646461	8.461192131	6.892159939
0.275981873	8.455350876	10.519919395
1.821233273	8.421794891	14.102534294
4.714686394	7.479999065	15.897046089
4.123764038	2.823067904	17.424839020
4.649536610	4.602709770	15.254326820
6.583897591	0.855622470	1.739840031
7.998667717	0.855622470	5.269619942
9.414875031	0.849487305	8.869804382
0.663733006	0.861832798	12.599900246
4.382317543	2.757042646	2.622289896
5.797186852	2.757042646	6.152059555
7.164693832	2.760585546	9.673459053
8.538176537	2.762811184	13.204102516
1.473447323	2.757042646	1.739840031
2.888217449	2.757042646	5.269619942
4.288507938	2.754093885	8.858341217
5.761153221	2.743087292	12.507046700
9.492767334	0.855622470	2.622289896
0.686737418	0.855622470	6.152059555
2.035506487	0.850520015	9.701925278
3.455372334	0.827244043	13.199974060
6.583897591	4.658352375	1.739840031
7.998667717	4.658352375	5.269619942
9.438307762	4.656955719	8.864246368
0.690079212	4.659110069	12.608865738
4.382317543	6.559772491	2.622289896
5.797186852	6.559772491	6.152059555
7.168609142	6.556813240	9.669970512
8.533386230	6.551184177	13.198142052

1.473447323	6.559772491	1.739840031
2.888217449	6.559772491	5.269619942
4.286425591	6.556180000	8.856201172
5.747887135	6.537181854	12.489993095
9.492767334	4.658352375	2.622289896
0.686737418	4.658352375	6.152059555
2.042328358	4.654408455	9.761119843
3.473530054	4.637199879	13.321447372
6.583897591	8.461192131	1.739840031
7.998667717	8.461192131	5.269619942
9.425557137	8.465350151	8.869377136
0.679973722	8.450173378	12.614016533
4.382317543	10.362502098	2.622289896
5.797186852	10.362502098	6.152059555
7.144721985	10.358964920	9.696566582
8.567564011	10.353792191	13.206128120
1.473447323	10.362502098	1.739840031
2.888217449	10.362502098	5.269619942
4.300650120	10.357125282	8.871052742
5.790951729	10.345060349	12.597533226
9.492767334	8.461192131	2.622289896
0.686737418	8.461192131	6.152059555
2.035588980	8.461023331	9.728178978
3.464608908	8.462114334	13.256641388

G3

3.514827251	5.738251686	17.782806396
5.344711304	4.169691086	18.130846024
4.950529575	6.798480034	17.819997787
2.796875477	6.953481197	15.975337982
6.836639881	4.609929085	17.255140305
4.342170238	3.590452433	15.662006378
6.300968647	2.925366640	15.732681274
5.672514439	5.853085041	15.604763031
4.233748436	6.293612957	17.152292252
5.796076298	4.274286747	17.132562637
5.002232552	5.308723927	16.283037186
4.737797260	0.855622470	2.479939699
6.152677536	0.855622470	6.009709835
7.564066887	0.853785515	9.599950790
9.064882278	0.854129374	13.443273544
6.228307724	2.757042646	1.882519841
7.643187523	2.757042646	5.412300110
9.048086166	2.755966187	8.975993156
0.270824581	2.751838207	12.592472076
8.429887772	0.855622470	1.000079989
9.844667435	0.855622470	4.529860020
1.034220815	0.858097017	8.087823868
2.511271715	0.827134490	11.654875755
2.536317587	2.757042646	3.362380028
3.951197147	2.757042646	6.892159939

5.386415958	2.756311178	10.504948616
6.917165756	2.771978378	14.067418098
9.848246574	2.757042646	2.479939699
1.042227387	2.757042646	6.009709835
2.456022263	2.753357172	9.630723000
3.955386639	2.725627422	13.428270340
1.117857456	0.855622470	1.882519841
2.532737255	0.855622470	5.412300110
3.929702759	0.854889035	8.974346161
5.393474579	0.847499967	12.577053070
3.319437265	2.757042646	1.000079989
4.734217644	2.757042646	4.529860020
6.137325287	2.754251719	8.086638451
7.619640827	2.747845173	11.641421318
7.646767139	0.855622470	3.362380028
9.061646461	0.855622470	6.892159939
0.271810442	0.851298273	10.507345200
1.816543341	0.859673321	14.072872162
4.737797260	4.658352375	2.479939699
6.152677536	4.658352375	6.009709835
7.566624165	4.655055046	9.592482567
9.048319817	4.658116817	13.422624588
6.228307724	6.559772491	1.882519841
7.643187523	6.559772491	5.412300110
9.051270485	6.556521416	8.971074104
0.271258444	6.560716629	12.565611839
8.429887772	4.658352375	1.000079989
9.844667435	4.658352375	4.529860020
1.045953512	4.657839775	8.102745056
2.522002459	4.634312630	11.718541145
2.536317587	6.559772491	3.362380028
3.951197147	6.559772491	6.892159939
5.387661934	6.554557323	10.492365837
6.914341927	6.557711601	14.027347565
9.848246574	6.559772491	2.479939699
1.042227387	6.559772491	6.009709835
2.459358931	6.557973862	9.647624969
3.948092937	6.566150188	13.396790504
1.117857456	4.658352375	1.882519841
2.532737255	4.658352375	5.412300110
3.926616669	4.656159401	8.980321884
5.369451046	4.650401592	12.582795143
3.319437265	6.559772491	1.000079989
4.734217644	6.559772491	4.529860020
6.136638641	6.557550430	8.080549240
7.624021530	6.559981346	11.619880676
7.646767139	4.658352375	3.362380028
9.061646461	4.658352375	6.892159939
0.270178169	4.654652596	10.510192871
1.732773781	4.691110611	14.092254639
4.737797260	8.461192131	2.479939699

6.152677536	8.461192131	6.009709835
7.564397812	8.459016800	9.592139244
9.067942619	8.454719543	13.441402435
6.228307724	10.362502098	1.882519841
7.643187523	10.362502098	5.412300110
9.048389435	10.360644341	8.975025177
0.284632355	10.358567238	12.606078148
8.429887772	8.461192131	1.000079989
9.844667435	8.461192131	4.529860020
1.038154364	8.452957153	8.091664314
2.517425537	8.499851227	11.673867226
2.536317587	10.362502098	3.362380028
3.951197147	10.362502098	6.892159939
5.386361122	10.358855247	10.499942780
6.930589199	10.358183861	14.069006920
9.848246574	10.362502098	2.479939699
1.042227387	10.362502098	6.009709835
2.450153112	10.360387802	9.603080750
3.971252441	10.359102249	13.470208168
1.117857456	8.461192131	1.882519841
2.532737255	8.461192131	5.412300110
3.928273678	8.457467079	8.975515366
5.384651661	8.460242271	12.574939728
3.319437265	10.362502098	1.000079989
4.734217644	10.362502098	4.529860020
6.142183304	10.363082886	8.084700584
7.629513741	10.355597496	11.649987221
7.646767139	8.461192131	3.362380028
9.061646461	8.461192131	6.892159939
0.274122566	8.463392258	10.509098053
1.806792855	8.418274879	14.084722519
4.072374344	4.536400318	15.470500946
3.578938007	7.330820084	16.418832779
5.724588871	2.971387863	16.541828156
6.583897591	0.855622470	1.739840031
7.998667717	0.855622470	5.269619942
9.409900665	0.851326346	8.869084358
0.664314151	0.853283465	12.593292236
4.382317543	2.757042646	2.622289896
5.797186852	2.757042646	6.152059555
7.156079292	2.761624813	9.682047844
8.553135872	2.758512974	13.186351776
1.473447323	2.757042646	1.739840031
2.888217449	2.757042646	5.269619942
4.288352966	2.756189108	8.863796234
5.743074417	2.764005899	12.538677216
9.492767334	0.855622470	2.622289896
0.686737418	0.855622470	6.152059555
2.032319069	0.851637661	9.700797081
3.448267698	0.835627437	13.204656601
6.583897591	4.658352375	1.739840031

7.998667717	4.658352375	5.269619942
9.437753677	4.655585766	8.853549957
0.670919776	4.658263206	12.563007355
4.382317543	6.559772491	2.622289896
5.797186852	6.559772491	6.152059555
7.159265518	6.551005363	9.673435211
8.548233986	6.554526329	13.174003601
1.473447323	6.559772491	1.739840031
2.888217449	6.559772491	5.269619942
4.288154125	6.556198597	8.863843918
5.752971649	6.550775051	12.527662277
9.492767334	4.658352375	2.622289896
0.686737418	4.658352375	6.152059555
2.051104069	4.655117989	9.769421577
3.436359882	4.666723728	13.376636505
6.583897591	8.461192131	1.739840031
7.998667717	8.461192131	5.269619942
9.414071083	8.461792946	8.866529465
0.666035831	8.452416420	12.591473579
4.382317543	10.362502098	2.622289896
5.797186852	10.362502098	6.152059555
7.144778728	10.359290123	9.692279816
8.561324120	10.357762337	13.202559471
1.473447323	10.362502098	1.739840031
2.888217449	10.362502098	5.269619942
4.295648098	10.359617233	8.868408203
5.781162262	10.356539726	12.587348938
9.492767334	8.461192131	2.622289896
0.686737418	8.461192131	6.152059555
2.034433603	8.460404396	9.711369514
3.449056625	8.470003128	13.234069824

G4

2.878121138	5.398086071	16.431497574
2.954910517	7.922908783	16.536809921
5.436467171	5.206390858	15.166246414
5.472790718	8.002182007	15.149718285
4.159085274	4.474325657	17.264770508
4.379327297	8.721591949	17.265077591
4.556194305	5.860610008	18.995452881
5.667670250	6.556775093	17.032415390
3.964963675	5.304013729	16.560709000
4.051670551	7.916439056	16.589073181
4.563481331	6.587153435	17.158630371
4.737797260	0.855622470	2.479939699
6.152677536	0.855622470	6.009709835
7.561130047	0.853195190	9.596252441
9.062591553	0.850819409	13.447294235
6.228307724	2.757042646	1.882519841
7.643187523	2.757042646	5.412300110
9.050888062	2.754663944	8.975108147

0.279396862	2.758810282	12.624915123
8.429887772	0.855622470	1.000079989
9.844667435	0.855622470	4.529860020
1.029501319	0.854165018	8.086235046
2.499695778	0.853813708	11.643054008
2.536317587	2.757042646	3.362380028
3.951197147	2.757042646	6.892159939
5.388654232	2.759181261	10.496865273
6.921789646	2.762392044	14.066983223
9.848246574	2.757042646	2.479939699
1.042227387	2.757042646	6.009709835
2.448364258	2.752990961	9.602512360
3.964985609	2.726975441	13.417260170
1.117857456	0.855622470	1.882519841
2.532737255	0.855622470	5.412300110
3.931784868	0.853268385	8.979828835
5.405942917	0.851854980	12.561094284
3.319437265	2.757042646	1.000079989
4.734217644	2.757042646	4.529860020
6.140199661	2.756957293	8.083539009
7.633327484	2.752945662	11.643420219
7.646767139	0.855622470	3.362380028
9.061646461	0.855622470	6.892159939
0.266721785	0.851763487	10.509758949
1.823765397	0.850509465	14.067561150
4.737797260	4.658352375	2.479939699
6.152677536	4.658352375	6.009709835
7.568053722	4.655208111	9.594067574
9.048782349	4.662983894	13.459672928
6.228307724	6.559772491	1.882519841
7.643187523	6.559772491	5.412300110
9.050949097	6.557355404	8.960876465
0.258418173	6.555325508	12.608595848
8.429887772	4.658352375	1.000079989
9.844667435	4.658352375	4.529860020
1.041143298	4.663710594	8.102705002
2.492331982	4.571835041	11.702584267
2.536317587	6.559772491	3.362380028
3.951197147	6.559772491	6.892159939
5.397074699	6.556952477	10.476796150
6.849038124	6.553986549	14.051714897
9.848246574	6.559772491	2.479939699
1.042227387	6.559772491	6.009709835
2.462830305	6.556862354	9.669223785
3.878021717	6.554963112	13.121415138
1.117857456	4.658352375	1.882519841
2.532737255	4.658352375	5.412300110
3.923671007	4.659556389	8.983359337
5.399454594	4.643957138	12.580770493
3.319437265	6.559772491	1.000079989
4.734217644	6.559772491	4.529860020

6.136344910	6.557635307	8.079002380
7.626744747	6.556487083	11.626213074
7.646767139	4.658352375	3.362380028
9.061646461	4.658352375	6.892159939
0.261938304	4.663756371	10.520523071
1.803502083	4.693534374	14.115866661
4.737797260	8.461192131	2.479939699
6.152677536	8.461192131	6.009709835
7.567211151	8.459015846	9.593388557
9.048066139	8.447416306	13.458852768
6.228307724	10.362502098	1.882519841
7.643187523	10.362502098	5.412300110
9.050694466	10.359574318	8.975069046
0.278872967	10.351489067	12.624592781
8.429887772	8.461192131	1.000079989
9.844667435	8.461192131	4.529860020
1.041721344	8.452306747	8.102837563
2.492365837	8.538837433	11.702016830
2.536317587	10.362502098	3.362380028
3.951197147	10.362502098	6.892159939
5.388649464	10.354004860	10.496075630
6.919241905	10.342427254	14.065254211
9.848246574	10.362502098	2.479939699
1.042227387	10.362502098	6.009709835
2.448412418	10.361021996	9.603388786
3.963485003	10.384645462	13.411038399
1.117857456	8.461192131	1.882519841
2.532737255	8.461192131	5.412300110
3.923088312	8.454886436	8.982575417
5.399657726	8.466790199	12.578931808
3.319437265	10.362502098	1.000079989
4.734217644	10.362502098	4.529860020
6.139843941	10.359388351	8.082978249
7.633244038	10.361209869	11.642144203
7.646767139	8.461192131	3.362380028
9.061646461	8.461192131	6.892159939
0.262047559	8.447622299	10.520292282
1.803918839	8.416780472	14.115725517
4.496943474	4.939092636	15.274882317
4.514542103	8.186245918	15.257291794
4.220204353	6.657824993	18.549278259
6.583897591	0.855622470	1.739840031
7.998667717	0.855622470	5.269619942
9.407732010	0.852539599	8.869929314
0.659246385	0.850709081	12.596632004
4.382317543	2.757042646	2.622289896
5.797186852	2.757042646	6.152059555
7.148939133	2.758529186	9.686235428
8.553907394	2.759598255	13.200990677
1.473447323	2.757042646	1.739840031
2.888217449	2.757042646	5.269619942

4.290732384	2.756933689	8.866306305
5.775812149	2.750432968	12.564804077
9.492767334	0.855622470	2.622289896
0.686737418	0.855622470	6.152059555
2.029926538	0.852908552	9.696352005
3.450411558	0.851603508	13.181401253
6.583897591	4.658352375	1.739840031
7.998667717	4.658352375	5.269619942
9.431354523	4.648366451	8.864669800
0.675985873	4.664153099	12.607398987
4.382317543	6.559772491	2.622289896
5.797186852	6.559772491	6.152059555
7.180859089	6.557323456	9.658594131
8.505038261	6.555269241	13.205701828
1.473447323	6.559772491	1.739840031
2.888217449	6.559772491	5.269619942
4.276074409	6.556812763	8.848629951
5.716056347	6.555464745	12.441252708
9.492767334	4.658352375	2.622289896
0.686737418	4.658352375	6.152059555
2.034905195	4.652708530	9.754590988
3.460802078	4.640349388	13.294089317
6.583897591	8.461192131	1.739840031
7.998667717	8.461192131	5.269619942
9.431888580	8.464956284	8.864281654
0.676172972	8.446347237	12.606505394
4.382317543	10.362502098	2.622289896
5.797186852	10.362502098	6.152059555
7.149321079	10.355834007	9.685486794
8.552567482	10.351428032	13.200750351
1.473447323	10.362502098	1.739840031
2.888217449	10.362502098	5.269619942
4.289950848	10.357397079	8.865614891
5.774343014	10.360803604	12.560397148
9.492767334	8.461192131	2.622289896
0.686737418	8.461192131	6.152059555
2.035356760	8.460629463	9.754814148
3.459749460	8.469467163	13.294810295

G5

4.755057812	8.593659401	17.250896454
3.219117641	7.890016079	16.672000885
5.552834034	7.769884109	15.007293701
5.104313374	7.137247562	18.911256790
5.869213581	6.324340343	16.805860519
2.960824251	5.437873363	16.445392609
4.143780708	4.413040161	17.305564880
5.373054981	4.369568825	15.307795525
4.312800884	7.819124222	16.596000671
4.802158833	6.444458961	17.085634232
4.030214310	5.219786644	16.565731049

4.737797260	0.855622470	2.479939699
6.152677536	0.855622470	6.009709835
7.562581062	0.851366282	9.590424538
9.058281898	0.857918262	13.452120781
6.228307724	2.757042646	1.882519841
7.643187523	2.757042646	5.412300110
9.053654671	2.754536867	8.975055695
0.270025641	2.764385700	12.627264023
8.429887772	0.855622470	1.000079989
9.844667435	0.855622470	4.529860020
1.031394362	0.851665735	8.087355614
2.499454498	0.845593750	11.647319794
2.536317587	2.757042646	3.362380028
3.951197147	2.757042646	6.892159939
5.391554832	2.752567530	10.493997574
6.902897358	2.804552794	14.067367554
9.848246574	2.757042646	2.479939699
1.042227387	2.757042646	6.009709835
2.449659586	2.752354860	9.608490944
3.967744112	2.721821547	13.395649910
1.117857456	0.855622470	1.882519841
2.532737255	0.855622470	5.412300110
3.927786827	0.854061544	8.973860741
5.410427570	0.846771777	12.565176964
3.319437265	2.757042646	1.000079989
4.734217644	2.757042646	4.529860020
6.139621258	2.755102158	8.081956863
7.634913921	2.741000891	11.641837120
7.646767139	0.855622470	3.362380028
9.061646461	0.855622470	6.892159939
0.266481757	0.854961872	10.511948586
1.828079939	0.861819625	14.071676254
4.737797260	4.658352375	2.479939699
6.152677536	4.658352375	6.009709835
7.566671848	4.652567863	9.586912155
9.042996407	4.672344685	13.451846123
6.228307724	6.559772491	1.882519841
7.643187523	6.559772491	5.412300110
9.053210258	6.558013439	8.963099480
0.269756496	6.563757896	12.611733437
8.429887772	4.658352375	1.000079989
9.844667435	4.658352375	4.529860020
1.042357326	4.662237644	8.102450371
2.491809368	4.574418545	11.699415207
2.536317587	6.559772491	3.362380028
3.951197147	6.559772491	6.892159939
5.396730423	6.556658268	10.472862244
6.860533237	6.624257088	14.031040192
9.848246574	6.559772491	2.479939699
1.042227387	6.559772491	6.009709835
2.462810993	6.558207989	9.671667099

3.888323307	6.544180393	13.162809372
1.117857456	4.658352375	1.882519841
2.532737255	4.658352375	5.412300110
3.922405958	4.658751965	8.982058525
5.398571491	4.642957687	12.561180115
3.319437265	6.559772491	1.000079989
4.734217644	6.559772491	4.529860020
6.135211468	6.557167053	8.076175690
7.628921986	6.541077137	11.615516663
7.646767139	4.658352375	3.362380028
9.061646461	4.658352375	6.892159939
0.261346281	4.666702747	10.520025253
1.799131870	4.693594456	14.110342026
4.737797260	8.461192131	2.479939699
6.152677536	8.461192131	6.009709835
7.568553448	8.458287239	9.593791962
9.060197830	8.456255913	13.453213692
6.228307724	10.362502098	1.882519841
7.643187523	10.362502098	5.412300110
9.054197311	10.358048439	8.976558685
0.287865311	10.360319138	12.630959511
8.429887772	8.461192131	1.000079989
9.844667435	8.461192131	4.529860020
1.044326305	8.453116417	8.107276917
2.505465508	8.529717445	11.719587326
2.536317587	10.362502098	3.362380028
3.951197147	10.362502098	6.892159939
5.390006065	10.363681793	10.493863106
6.925772190	10.339871407	14.063515663
9.848246574	10.362502098	2.479939699
1.042227387	10.362502098	6.009709835
2.451371193	10.361042976	9.612944603
3.972303391	10.380711555	13.437323570
1.117857456	8.461192131	1.882519841
2.532737255	8.461192131	5.412300110
3.924077749	8.456547737	8.984195709
5.386314869	8.465889931	12.547091484
3.319437265	10.362502098	1.000079989
4.734217644	10.362502098	4.529860020
6.140999317	10.355464935	8.082060814
7.635025978	10.358798981	11.641251564
7.646767139	8.461192131	3.362380028
9.061646461	8.461192131	6.892159939
0.267569423	8.454770088	10.526758194
1.816029549	8.428185463	14.133888245
4.657447338	8.124174118	15.237148285
4.640012264	6.380865097	18.511133194
4.477873325	4.763096809	15.278481483
6.583897591	0.855622470	1.739840031
7.998667717	0.855622470	5.269619942
9.410009384	0.850275457	8.870923042

0.660968661	0.859766066	12.603940010
4.382317543	2.757042646	2.622289896
5.797186852	2.757042646	6.152059555
7.155529976	2.756891966	9.679433823
8.542761803	2.764632463	13.206089973
1.473447323	2.757042646	1.739840031
2.888217449	2.757042646	5.269619942
4.287509441	2.756094933	8.861218452
5.767869473	2.748690605	12.535029411
9.492767334	0.855622470	2.622289896
0.686737418	0.855622470	6.152059555
2.028853893	0.852537453	9.699352264
3.454203606	0.845036089	13.183772087
6.583897591	4.658352375	1.739840031
7.998667717	4.658352375	5.269619942
9.432268143	4.650541306	8.864524841
0.672912240	4.668439865	12.603306770
4.382317543	6.559772491	2.622289896
5.797186852	6.559772491	6.152059555
7.179138184	6.557695389	9.655036926
8.514019012	6.565925598	13.191190720
1.473447323	6.559772491	1.739840031
2.888217449	6.559772491	5.269619942
4.275559902	6.559746265	8.850168228
5.720826626	6.549326420	12.443520546
9.492767334	4.658352375	2.622289896
0.686737418	4.658352375	6.152059555
2.037025213	4.654030323	9.754308701
3.457309246	4.640429974	13.292448044
6.583897591	8.461192131	1.739840031
7.998667717	8.461192131	5.269619942
9.436867714	8.465193748	8.866831779
0.692850828	8.454687119	12.620773315
4.382317543	10.362502098	2.622289896
5.797186852	10.362502098	6.152059555
7.149055958	10.353845596	9.685699463
8.557656288	10.354528427	13.197490692
1.473447323	10.362502098	1.739840031
2.888217449	10.362502098	5.269619942
4.291888714	10.358682632	8.866498947
5.777963161	10.353209496	12.566329002
9.492767334	8.461192131	2.622289896
0.686737418	8.461192131	6.152059555
2.036927938	8.463516235	9.764455795
3.474529982	8.466233253	13.317899704

CONTCAR A001
H 8 C 3 O 93 Ti 45
1.0

11.4083003998	0.0000000000	0.0000000000
0.0000000000	11.4083003998	0.0000000000
0.0000000000	0.0000000000	30.2532005310

H	C	O	Ti
8	3	93	45

G1

6.308906078	5.974983692	14.441725731
6.096605778	7.721545219	15.970299721
8.791653633	6.517875671	14.394477844
7.264183998	5.868057728	12.110871315
5.001965523	8.316718102	14.022257805
3.887730122	6.943639755	12.769242287
8.435760498	7.861881733	13.281206131
6.159445286	8.443182945	12.670866013
6.697640896	6.990122318	14.234314919
8.097239494	6.867582798	13.619994164
5.664321899	7.742282867	13.358376503
2.765438318	2.908519506	5.039564133
2.757042646	1.007697463	3.423067570
2.757042646	2.909117460	1.051217556
2.941150665	2.897270203	10.870518684
2.758712530	1.006755352	7.454862595
0.855622470	1.007697463	0.285207510
1.066655159	1.003416061	9.878976822
0.753807247	2.898115396	8.312225342
0.858112752	1.007934570	5.882320881
0.855622470	2.909117460	2.657057762
6.558036804	2.909737825	5.042037010
6.559772491	1.007697463	3.423067570
6.559772491	2.909117460	1.051217556
6.752451897	2.933914423	10.865754128
6.554159164	0.998762846	7.458612919
4.658352375	1.007697463	0.285207510
4.868131638	1.008828521	9.893864632
4.565778255	2.915471792	8.333597183
4.662634850	1.009638548	5.889926434
4.658352375	2.909117460	2.657057762
10.363177299	2.906194210	5.038410187
10.362502098	1.007697463	3.423067570
10.362502098	2.909117460	1.051217556
10.558053017	2.891991138	10.855361938
10.353813171	1.000352144	7.457201958
8.461192131	1.007697463	0.285207510
8.650670052	1.000060201	9.897070885
8.349409103	2.897908211	8.326764107
8.460893631	1.007216334	5.889255524
8.461192131	2.909117460	2.657057762

2.772535801	6.712508678	5.040470123
2.757042646	4.810537338	3.423067570
2.757042646	6.711847782	1.051217556
2.837431669	6.703791142	10.873097420
2.756209612	4.801089287	7.452247143
0.855622470	4.810537338	0.285207510
1.049005508	4.797599316	9.861028671
0.757087946	6.709441185	8.292195320
0.863604784	4.813955307	5.869624615
0.855622470	6.711847782	2.657057762
6.558903694	6.714185715	5.041724205
6.559772491	4.810537338	3.423067570
6.559772491	6.711847782	1.051217556
6.716114044	6.713472366	10.614234924
6.560456753	4.840405464	7.471166134
4.658352375	4.810537338	0.285207510
4.804230213	4.820197105	9.914625168
4.532209873	6.711571693	8.351717949
4.666523457	4.811301708	5.897922516
4.658352375	6.711847782	2.657057762
10.358033180	6.712489128	5.042171478
10.362502098	4.810537338	3.423067570
10.362502098	6.711847782	1.051217556
10.594412804	6.729562759	10.859443665
10.366184235	4.773778915	7.441140652
8.461192131	4.810537338	0.285207510
8.694792747	4.795925617	9.868123055
8.477509499	6.702345848	8.320641518
8.461382866	4.805570126	5.881172180
8.461192131	6.711847782	2.657057762
2.764274836	10.515294075	5.039938927
2.757042646	8.613267899	3.423067570
2.757042646	10.514687538	1.051217556
2.945444107	10.523885727	10.882974625
2.754371643	8.619426727	7.452741146
0.855622470	8.613267899	0.285207510
1.064940572	8.623638153	9.861613274
0.752072871	10.519932747	8.313137054
0.860157847	8.610369682	5.872587204
0.855622470	10.514687538	2.657057762
6.560073853	10.511431694	5.045270920
6.559772491	8.613267899	3.423067570
6.559772491	10.514687538	1.051217556
6.754443645	10.480303764	10.886447906
6.557939529	8.576612473	7.481285572
4.658352375	8.613267899	0.285207510
4.799107552	8.606676102	9.915392876
4.560441971	10.511075974	8.333222389
4.663724422	8.616077423	5.903311253
4.658352375	10.514687538	2.657057762
10.360610962	10.515137672	5.039239407

10.362502098	8.613267899	3.423067570
10.362502098	10.514687538	1.051217556
10.554506302	10.513530731	10.857682228
10.365411758	8.640586853	7.451105118
8.461192131	8.613267899	0.285207510
8.682071686	8.597685814	9.907707214
8.348738670	10.502431870	8.336229324
8.461836815	8.612219810	5.900235176
8.461192131	10.514687538	2.657057762
8.159031868	5.914250851	12.538746834
6.919843674	7.720568657	15.451885223
4.838728428	6.841151714	12.562856674
2.757042646	2.909117460	3.040067673
2.763526201	1.007987380	5.447085381
0.881303489	1.005708933	7.890697479
0.855622470	2.909117460	0.668217480
0.835316956	2.902299643	10.260582924
6.559772491	2.909117460	3.040067673
6.562828541	1.007145166	5.446365833
4.685243607	1.009304166	7.904405594
4.658352375	2.909117460	0.668217480
4.629097939	2.910231352	10.273813248
10.362502098	2.909117460	3.040067673
10.361959457	1.007449627	5.448055267
8.483760834	1.000403166	7.905995369
8.461192131	2.909117460	0.668217480
8.444398880	2.906475067	10.271790504
2.757042646	6.711847782	3.040067673
2.765964508	4.810280800	5.447885990
0.880159080	4.803263187	7.889867783
0.855622470	6.711847782	0.668217480
0.862918556	6.710084438	10.241586685
6.559772491	6.711847782	3.040067673
6.564648628	4.814089775	5.450210094
4.683310032	4.832108974	7.912421227
4.658352375	6.711847782	0.668217480
4.593144417	6.709455013	10.345412254
10.362502098	6.711847782	3.040067673
10.368345261	4.808985233	5.444766521
8.491684914	4.809638023	7.897356033
8.461192131	6.711847782	0.668217480
8.501995087	6.691135883	10.307365417
2.757042646	10.514687538	3.040067673
2.761905909	8.613486290	5.447579861
0.880645156	8.615179062	7.891937256
0.855622470	10.514687538	0.668217480
0.833275080	10.516585350	10.259359360
6.559772491	10.514687538	3.040067673
6.559772491	8.613267899	5.474937439
4.676720619	8.591726303	7.920475006
4.658352375	10.514687538	0.668217480

4.628605843	10.519215584	10.271441460
10.362502098	10.514687538	3.040067673
10.363584518	8.614174843	5.447574139
8.496726036	8.588661194	7.913005352
8.461192131	10.514687538	0.668217480
8.442497253	10.503558159	10.269619942

G2

5.733297348	5.120505810	15.762681007
5.361520290	5.027577877	13.251531601
4.119130135	5.883205891	15.875088692
3.825058222	5.961191654	13.322262764
6.541695595	6.979340553	14.341893196
6.885639191	6.754165649	11.923698425
5.349760056	7.722557545	16.794389725
5.064633846	8.659130096	13.851712227
5.202940464	6.083261013	15.770164490
4.922204018	6.041128159	13.261399269
5.453662395	6.823573112	14.458845139
2.764263868	2.906936884	5.038487911
2.757042646	1.007697463	3.423067570
2.757042646	2.909117460	1.051217556
2.918205261	2.926441193	10.851985931
2.758527994	1.009315014	7.461683273
0.855622470	1.007697463	0.285207510
1.054876089	1.009650826	9.875531197
0.743757367	2.898734808	8.302622795
0.853176236	1.007443786	5.883190155
0.855622470	2.909117460	2.657057762
6.552432060	2.911383390	5.043388844
6.559772491	1.007697463	3.423067570
6.559772491	2.909117460	1.051217556
6.712702274	2.956314087	10.836598396
6.546970367	1.011087418	7.466482162
4.658352375	1.007697463	0.285207510
4.826545715	1.011842728	9.905299187
4.581941605	2.932522058	8.342500687
4.658188820	1.008421063	5.897674084
4.658352375	2.909117460	2.657057762
10.347345352	2.907079935	5.039674759
10.362502098	1.007697463	3.423067570
10.362502098	2.909117460	1.051217556
10.547060013	2.903531790	10.843043327
10.342676163	1.010077000	7.460400105
8.461192131	1.007697463	0.285207510
8.624813080	1.012725115	9.908249855
8.340767860	2.923616171	8.337812424
8.450830460	1.008007407	5.896694660
8.461192131	2.909117460	2.657057762
2.763941288	6.712563038	5.042612553
2.757042646	4.810537338	3.423067570

2.757042646	6.711847782	1.051217556
2.631102324	6.696958542	10.991821289
2.746476173	4.776857853	7.443049431
0.855622470	4.810537338	0.285207510
1.004007578	4.808118820	9.831748962
0.804653347	6.714087486	8.255961418
0.850744784	4.805294991	5.853599072
0.855622470	6.711847782	2.657057762
6.558493614	6.714845657	5.038188934
6.559772491	4.810537338	3.423067570
6.559772491	6.711847782	1.051217556
7.893860817	6.733238220	11.860920906
6.550333023	4.887926579	7.483378887
4.658352375	4.810537338	0.285207510
4.762941360	4.871433735	9.923338890
4.466709137	6.716720104	8.336394310
4.662902832	4.814540863	5.894436836
4.658352375	6.711847782	2.657057762
10.343655586	6.712575912	5.040741444
10.362502098	4.810537338	3.423067570
10.362502098	6.711847782	1.051217556
10.681847572	6.711479664	11.003949165
10.352746010	4.804051399	7.444396973
8.461192131	4.810537338	0.285207510
8.682944298	4.825971127	9.899488449
8.497813225	6.716249466	8.380147934
8.447995186	4.808157921	5.893819332
8.461192131	6.711847782	2.657057762
2.765017271	10.516721725	5.038635731
2.757042646	8.613267899	3.423067570
2.757042646	10.514687538	1.051217556
2.928125858	10.500802040	10.849038124
2.746929407	8.654655457	7.446197510
0.855622470	8.613267899	0.285207510
1.012097359	8.617253304	9.836393356
0.745877922	10.528062820	8.306241035
0.852509737	8.617897034	5.855687141
0.855622470	10.514687538	2.657057762
6.551807880	10.511705399	5.046840668
6.559772491	8.613267899	3.423067570
6.559772491	10.514687538	1.051217556
6.719000340	10.477733612	10.841704369
6.553518295	8.545598030	7.488863468
4.658352375	8.613267899	0.285207510
4.787255287	8.558422089	9.925253868
4.584637642	10.497550011	8.343530655
4.661917210	8.609457016	5.900262356
4.658352375	10.514687538	2.657057762
10.349766731	10.517151833	5.039662361
10.362502098	8.613267899	3.423067570
10.362502098	10.514687538	1.051217556

10.551238060	10.526683807	10.842731476
10.357024193	8.627080917	7.446363926
8.461192131	8.613267899	0.285207510
8.693748474	8.608883858	9.898176193
8.342178345	10.509483337	8.339624405
8.452998161	8.618473053	5.897691250
8.461192131	10.514687538	2.657057762
5.698848724	6.815793991	16.885663986
5.278993130	6.707097530	12.037079811
4.789392471	8.093062401	14.593139648
2.757042646	2.909117460	3.040067673
2.766271591	1.008279324	5.451309681
0.874772966	1.009589076	7.883719921
0.855622470	2.909117460	0.668217480
0.824534535	2.904195070	10.258720398
6.559772491	2.909117460	3.040067673
6.554037094	1.007285953	5.452037334
4.679967403	1.011124849	7.929966450
4.658352375	2.909117460	0.668217480
4.615895748	2.935097456	10.273612976
10.362502098	2.909117460	3.040067673
10.348510742	1.008204103	5.453131199
8.473361969	1.012111068	7.922509193
8.461192131	2.909117460	0.668217480
8.417812347	2.928724289	10.272475243
2.757042646	6.711847782	3.040067673
2.765219688	4.808330536	5.444500446
0.861628413	4.787697792	7.853186607
0.855622470	6.711847782	0.668217480
0.919115126	6.712165833	10.185690880
6.559772491	6.711847782	3.040067673
6.557319641	4.815155506	5.448840618
4.668562412	4.857613564	7.915508270
4.658352375	6.711847782	0.668217480
4.332326889	6.725145817	10.413989067
10.362502098	6.711847782	3.040067673
10.345690727	4.809401512	5.444039822
8.467379570	4.850524426	7.920401096
8.461192131	6.711847782	0.668217480
8.936567307	6.716642380	10.401771545
2.757042646	10.514687538	3.040067673
2.763533115	8.615818024	5.443675041
0.864391088	8.639965057	7.857585907
0.855622470	10.514687538	0.668217480
0.829441428	10.523921013	10.261152267
6.559772491	10.514687538	3.040067673
6.559772491	8.613267899	5.474937439
4.666102886	8.572212219	7.914789677
4.658352375	10.514687538	0.668217480
4.625695229	10.501875877	10.273488045
10.362502098	10.514687538	3.040067673

10.348223686	8.615177155	5.443075180
8.477624893	8.581888199	7.925372601
8.461192131	10.514687538	0.668217480
8.423766136	10.506704330	10.273978233

G3

5.564337730	6.172114849	15.507253647
3.445087194	6.237421513	15.064791679
6.253148556	5.311686993	14.106376648
3.334106922	5.411890030	13.488678932
7.007267952	7.908005238	14.967654228
4.868959904	8.017436028	13.801685333
2.377039671	7.317203522	12.691322327
6.881026745	6.757395267	11.995521545
5.977998257	6.304493427	14.490972519
3.489360571	6.392546177	13.976808548
4.883507252	6.927180290	13.610378265
2.785452127	2.906955481	5.038492203
2.757042646	1.007697463	3.423067570
2.757042646	2.909117460	1.051217556
2.955488205	2.927513599	10.867920876
2.778503895	1.002330422	7.465615273
0.855622470	1.007697463	0.285207510
1.090993643	0.999352396	9.873738289
0.763776302	2.887651682	8.302398682
0.870783150	1.007093191	5.882900238
0.855622470	2.909117460	2.657057762
6.558299541	2.910685539	5.043670654
6.559772491	1.007697463	3.423067570
6.559772491	2.909117460	1.051217556
6.761766911	2.935892820	10.843960762
6.555989742	0.997114122	7.469954491
4.658352375	1.007697463	0.285207510
4.852896214	1.002501607	9.920715332
4.600891590	2.933229208	8.368594170
4.671325684	1.009194493	5.906396866
4.658352375	2.909117460	2.657057762
10.362196922	2.905222893	5.038688660
10.362502098	1.007697463	3.423067570
10.362502098	2.909117460	1.051217556
10.590817451	2.893516541	10.847579956
10.360171318	0.998881936	7.456364632
8.461192131	1.007697463	0.285207510
8.672655106	0.999849498	9.898865700
8.346631050	2.903533936	8.327058792
8.461074829	1.006800890	5.894340038
8.461192131	2.909117460	2.657057762
2.778408051	6.711826801	5.041777611
2.757042646	4.810537338	3.423067570
2.757042646	6.711847782	1.051217556
2.651904106	6.720483780	10.961706161

2.766680956	4.775988579	7.447604656
0.855622470	4.810537338	0.285207510
1.047244310	4.797829628	9.824040413
0.790202916	6.701550007	8.236011505
0.870434821	4.799322605	5.850515842
0.855622470	6.711847782	2.657057762
6.564756870	6.714096546	5.037647247
6.559772491	4.810537338	3.423067570
6.559772491	6.711847782	1.051217556
7.846607685	6.682118893	11.755772591
6.563919067	4.878323555	7.485727787
4.658352375	4.810537338	0.285207510
4.827612877	4.875737667	9.964993477
4.484988689	6.705429554	8.378638268
4.677950382	4.819999695	5.909929752
4.658352375	6.711847782	2.657057762
10.362324715	6.711603165	5.042193413
10.362502098	4.810537338	3.423067570
10.362502098	6.711847782	1.051217556
10.693014145	6.696810722	10.973485947
10.369194031	4.777931690	7.433929920
8.461192131	4.810537338	0.285207510
8.735303879	4.799907684	9.869276047
8.516299248	6.702217579	8.353955269
8.459888458	4.805408955	5.883957863
8.461192131	6.711847782	2.657057762
2.783959627	10.516034126	5.039438725
2.757042646	8.613267899	3.423067570
2.757042646	10.514687538	1.051217556
2.949122906	10.482619286	10.857161522
2.761054754	8.631658554	7.451258659
0.855622470	8.613267899	0.285207510
1.030974269	8.606133461	9.826382637
0.763718903	10.520077705	8.303175926
0.866830528	8.624020576	5.853028297
0.855622470	10.514687538	2.657057762
6.555999279	10.510910034	5.046818733
6.559772491	8.613267899	3.423067570
6.559772491	10.514687538	1.051217556
6.762242794	10.476140022	10.846681595
6.560488701	8.531684875	7.492372513
4.658352375	8.613267899	0.285207510
4.825096607	8.541527748	9.958249092
4.596899986	10.482262611	8.366126060
4.671439171	8.605147362	5.914735794
4.658352375	10.514687538	2.657057762
10.360886574	10.517263412	5.039348125
10.362502098	8.613267899	3.423067570
10.362502098	10.514687538	1.051217556
10.584439278	10.514767647	10.846764565
10.367465973	8.626893044	7.439019203

8.461192131	8.613267899	0.285207510
8.722186089	8.599214554	9.880731583
8.350536346	10.500351906	8.331676483
8.458822250	8.617731094	5.892079830
8.461192131	10.514687538	2.657057762
7.189575195	7.055720329	14.534569740
2.446609735	7.307615757	13.668571472
5.186201572	6.753030300	12.207607269
2.757042646	2.909117460	3.040067673
2.783884764	1.007569790	5.452092648
0.894044518	0.999731958	7.881125927
0.855622470	2.909117460	0.668217480
0.864293098	2.892524242	10.258736610
6.559772491	2.909117460	3.040067673
6.560087204	1.007008553	5.453457355
4.696203232	1.002376437	7.948767662
4.658352375	2.909117460	0.668217480
4.655089378	2.930325270	10.292063713
10.362502098	2.909117460	3.040067673
10.361305237	1.007408261	5.453392029
8.489150047	0.997776270	7.914783478
8.461192131	2.909117460	0.668217480
8.457732201	2.910029411	10.265086174
2.757042646	6.711847782	3.040067673
2.786905050	4.808029175	5.445446491
0.880093277	4.771010399	7.842881203
0.855622470	6.711847782	0.668217480
0.917987704	6.696462154	10.152976036
6.559772491	6.711847782	3.040067673
6.567778111	4.815069675	5.448689461
4.687046528	4.862957001	7.935683727
4.658352375	6.711847782	0.668217480
4.394037724	6.706539154	10.508905411
10.362502098	6.711847782	3.040067673
10.360720634	4.807878971	5.441554546
8.483003616	4.827148914	7.908032894
8.461192131	6.711847782	0.668217480
8.941551208	6.698391438	10.355878830
2.757042646	10.514687538	3.040067673
2.778896809	8.615063667	5.446053028
0.875383198	8.635045052	7.850322247
0.855622470	10.514687538	0.668217480
0.859624147	10.512177467	10.259334564
6.559772491	10.514687538	3.040067673
6.559772491	8.613267899	5.474937439
4.676484108	8.549674034	7.940754414
4.658352375	10.514687538	0.668217480
4.650497913	10.483482361	10.290290833
10.362502098	10.514687538	3.040067673
10.356987000	8.615290642	5.442947865
8.484734535	8.576434135	7.915054798

8.461192131	10.514687538	0.668217480
8.457819939	10.495640755	10.269056320

G4

3.183985233	4.744526863	13.123469353
5.867787361	4.146183968	12.972037315
3.419467688	5.124785900	14.853370667
5.624620914	4.354135990	14.732344627
2.449199677	6.875187397	12.856979370
7.654636860	6.201788902	12.543396950
7.380572319	5.872556686	14.644943237
5.124374390	6.847795010	14.231020927
3.457574129	5.545719624	13.836602211
5.888985634	4.872309208	13.795812607
4.896030903	6.009435654	13.542407036
2.776218891	2.904935360	5.038568974
2.757042646	1.007697463	3.423067570
2.757042646	2.909117460	1.051217556
2.933095694	2.922030449	10.864207268
2.768410206	0.986386836	7.462810516
0.855622470	1.007697463	0.285207510
1.075387478	1.002745271	9.869283676
0.749764800	2.886758804	8.293360710
0.861282766	1.008281469	5.878403664
0.855622470	2.909117460	2.657057762
6.551298618	2.910353899	5.043534279
6.559772491	1.007697463	3.423067570
6.559772491	2.909117460	1.051217556
6.739038944	2.943102598	10.851037025
6.548484802	0.998666584	7.472477913
4.658352375	1.007697463	0.285207510
4.836403847	0.988576055	9.920534134
4.580996513	2.912959576	8.349474907
4.664309025	1.003570676	5.905665398
4.658352375	2.909117460	2.657057762
10.347132683	2.906079292	5.038897038
10.362502098	1.007697463	3.423067570
10.362502098	2.909117460	1.051217556
10.563889503	2.900314093	10.839673042
10.348584175	1.005989313	7.455382347
8.461192131	1.007697463	0.285207510
8.654254913	1.010194182	9.901860237
8.341033936	2.921005487	8.337430000
8.451869011	1.009901524	5.895673752
8.461192131	2.909117460	2.657057762
2.766610384	6.711417198	5.042669773
2.757042646	4.810537338	3.423067570
2.757042646	6.711847782	1.051217556
2.616560221	6.720547676	10.964780807
2.751866817	4.761078835	7.440295219
0.855622470	4.810537338	0.285207510

1.023478508	4.796270847	9.815444946
0.800593019	6.703832626	8.224239349
0.853823900	4.799249172	5.845206738
0.855622470	6.711847782	2.657057762
6.562253952	6.714713097	5.037917137
6.559772491	4.810537338	3.423067570
6.559772491	6.711847782	1.051217556
7.862371922	6.814420700	11.777487755
6.547433376	4.875362396	7.485186100
4.658352375	4.810537338	0.285207510
4.792798996	4.853672028	9.934823990
4.456573486	6.698585510	8.362362862
4.665955067	4.819005489	5.896866798
4.658352375	6.711847782	2.657057762
10.346742630	6.711753368	5.042018414
10.362502098	4.810537338	3.423067570
10.362502098	6.711847782	1.051217556
10.697078705	6.685769081	10.990559578
10.353057861	4.787283897	7.441037178
8.461192131	4.810537338	0.285207510
8.698246956	4.831193447	9.902297974
8.490802765	6.712929726	8.345662117
8.446719170	4.807629108	5.893502712
8.461192131	6.711847782	2.657057762
2.779842615	10.515243530	5.037969112
2.757042646	8.613267899	3.423067570
2.757042646	10.514687538	1.051217556
2.933272123	10.473314285	10.852625847
2.754029751	8.645176888	7.449717999
0.855622470	8.613267899	0.285207510
0.999028206	8.610564232	9.818729401
0.746472895	10.523447037	8.298566818
0.861238182	8.624892235	5.844882488
0.855622470	10.514687538	2.657057762
6.549838066	10.511507034	5.046665668
6.559772491	8.613267899	3.423067570
6.559772491	10.514687538	1.051217556
6.758833408	10.474093437	10.837717056
6.554484844	8.536554337	7.493512630
4.658352375	8.613267899	0.285207510
4.825808525	8.518080711	9.978369713
4.590269089	10.465636253	8.373371124
4.667248249	8.597637177	5.917021275
4.658352375	10.514687538	2.657057762
10.351358414	10.518511772	5.039114475
10.362502098	8.613267899	3.423067570
10.362502098	10.514687538	1.051217556
10.577755928	10.529076576	10.859612465
10.362368584	8.624422073	7.432860851
8.461192131	8.613267899	0.285207510
8.752967834	8.610799789	9.871304512

8.348637581	10.509074211	8.329021454
8.452787399	8.624488831	5.890012741
8.461192131	10.514687538	2.657057762
5.014339447	6.512507439	12.224473953
7.266072273	5.304810047	13.861667633
2.530281544	6.617789268	13.800749779
2.757042646	2.909117460	3.040067673
2.776847601	1.005952358	5.450819969
0.883485675	0.998752773	7.874556065
0.855622470	2.909117460	0.668217480
0.839601636	2.895236254	10.246520996
6.559772491	2.909117460	3.040067673
6.553828239	1.007102370	5.452697754
4.686366558	0.985112548	7.944784641
4.658352375	2.909117460	0.668217480
4.628146648	2.912572861	10.268569946
10.362502098	2.909117460	3.040067673
10.352081299	1.007751822	5.452045918
8.479201317	1.006854653	7.916879654
8.461192131	2.909117460	0.668217480
8.439983368	2.924741268	10.264378548
2.757042646	6.711847782	3.040067673
2.774313450	4.806781292	5.443064690
0.864020169	4.774142742	7.836858273
0.855622470	6.711847782	0.668217480
0.920996308	6.701336384	10.147752762
6.559772491	6.711847782	3.040067673
6.554198265	4.814699173	5.449013710
4.667529583	4.856657505	7.929987431
4.658352375	6.711847782	0.668217480
4.382667065	6.688679218	10.523083687
10.362502098	6.711847782	3.040067673
10.345661163	4.808027744	5.442967415
8.464960098	4.849479198	7.913625717
8.461192131	6.711847782	0.668217480
8.959121704	6.725351810	10.369910240
2.757042646	10.514687538	3.040067673
2.776718378	8.614466667	5.445769787
0.870108068	8.636462212	7.843797207
0.855622470	10.514687538	0.668217480
0.847524405	10.515922546	10.254411697
6.559772491	10.514687538	3.040067673
6.559772491	8.613267899	5.474937439
4.672049522	8.532872200	7.941148281
4.658352375	10.514687538	0.668217480
4.639388561	10.463765144	10.292701721
10.362502098	10.514687538	3.040067673
10.354616165	8.615307808	5.439948559
8.480550766	8.581607819	7.910052299
8.461192131	10.514687538	0.668217480
8.454409599	10.505227089	10.265607834

G5

3.230672836	4.455562115	12.694347382
5.884195805	4.188488007	13.150871277
3.324470043	4.195796967	14.452521324
5.774757385	4.419126511	14.922243118
1.894255877	6.197901726	13.048334122
5.741086483	6.857665062	12.681334496
7.199762821	6.144461155	14.695053101
4.438663483	6.496132374	14.613905907
3.268750429	4.974391937	13.670876503
5.804259300	4.938858509	13.951920509
4.547727585	5.790831566	13.771712303
2.778998852	2.910549164	5.043185234
2.757042646	1.007697463	3.423067570
2.757042646	2.909117460	1.051217556
2.571639776	2.924546242	10.835203171
2.779996395	1.003036737	7.461953163
0.855622470	1.007697463	0.285207510
0.641607344	1.006369710	9.888175011
1.000334263	2.902376413	8.316784859
0.865819633	1.008704782	5.886051178
0.855622470	2.909117460	2.657057762
6.547211647	2.908641815	5.040656090
6.559772491	1.007697463	3.423067570
6.559772491	2.909117460	1.051217556
6.327985287	2.937777758	10.890853882
6.550813198	0.997771442	7.462199688
4.658352375	1.007697463	0.285207510
4.473843575	0.994404674	9.903456688
4.738090515	2.929075241	8.353254318
4.660646439	1.003107548	5.898894787
4.658352375	2.909117460	2.657057762
10.368659973	2.905439615	5.037634373
10.362502098	1.007697463	3.423067570
10.362502098	2.909117460	1.051217556
10.143704414	2.877658606	10.846309662
10.373687744	1.006091118	7.455215454
8.461192131	1.007697463	0.285207510
8.205207825	1.008210540	9.875713348
8.564897537	2.896712780	8.310351372
8.456359863	1.009874344	5.885217667
8.461192131	2.909117460	2.657057762
2.773033619	6.711860657	5.036200523
2.757042646	4.810537338	3.423067570
2.757042646	6.711847782	1.051217556
1.798782706	6.727483273	11.468943596
2.770859718	4.863620758	7.477284431
0.855622470	4.810537338	0.285207510
0.555432618	4.781437397	9.853517532
0.880113780	6.711068630	8.328272820

0.870555162	4.805843830	5.875016689
0.855622470	6.711847782	2.657057762
6.552345753	6.713368893	5.044919968
6.559772491	4.810537338	3.423067570
6.559772491	6.711847782	1.051217556
6.623987675	6.759802341	10.783486366
6.572538853	4.797188282	7.457150936
4.658352375	4.810537338	0.285207510
4.446488380	4.871627331	9.961133957
4.852663517	6.701736450	8.385115623
4.658609867	4.818948269	5.914106846
4.658352375	6.711847782	2.657057762
10.373531342	6.711911678	5.041889191
10.362502098	4.810537338	3.423067570
10.362502098	6.711847782	1.051217556
10.071634293	6.707137585	11.050391197
10.373884201	4.774150372	7.432229042
8.461192131	4.810537338	0.285207510
8.292727470	4.803690910	9.846096039
8.615383148	6.707967281	8.270989418
8.462634087	4.806848049	5.861975193
8.461192131	6.711847782	2.657057762
2.778607130	10.512066841	5.042878628
2.757042646	8.613267899	3.423067570
2.757042646	10.514687538	1.051217556
2.567937613	10.490950584	10.838386536
2.767162085	8.548839569	7.479984760
0.855622470	8.613267899	0.285207510
0.540614069	8.644157410	9.848779678
1.002746344	10.519069672	8.317447662
0.870017648	8.620365143	5.874668121
0.855622470	10.514687538	2.657057762
6.547995090	10.511645317	5.043799877
6.559772491	8.613267899	3.423067570
6.559772491	10.514687538	1.051217556
6.341687679	10.464893341	10.874784470
6.573436737	8.616723061	7.469773769
4.658352375	8.613267899	0.285207510
4.429937363	8.522777557	9.975377083
4.732815742	10.468259811	8.365856171
4.656137943	8.601587296	5.924815178
4.658352375	10.514687538	2.657057762
10.368181229	10.518803596	5.037538052
10.362502098	8.613267899	3.423067570
10.362502098	10.514687538	1.051217556
10.139757156	10.551882744	10.862750053
10.375113487	8.645411491	7.431129932
8.461192131	8.613267899	0.285207510
8.330823898	8.619090080	9.843944550
8.566958427	10.526872635	8.309359550
8.463553429	8.618229866	5.866451263

8.461192131	10.514687538	2.657057762
4.781318665	6.631879330	12.583282471
6.984562397	5.760662556	13.827508926
2.101056814	5.729183197	13.906265259
2.757042646	2.909117460	3.040067673
2.767034531	1.007736444	5.450865269
0.840230286	1.004427791	7.903096676
0.855622470	2.909117460	0.668217480
0.869227648	2.911810398	10.248915672
6.559772491	2.909117460	3.040067673
6.547924519	1.005071998	5.447508812
4.637705326	0.992997527	7.934344769
4.658352375	2.909117460	0.668217480
4.643909931	2.916284323	10.279348373
10.362502098	2.909117460	3.040067673
10.369256020	1.008039594	5.451760292
8.435457230	1.006741881	7.886413097
8.461192131	2.909117460	0.668217480
8.465937614	2.899776936	10.263237953
2.757042646	6.711847782	3.040067673
2.752954721	4.812086105	5.449376583
0.840115547	4.841862679	7.923093319
0.855622470	6.711847782	0.668217480
0.479991376	6.715647697	10.370894432
6.559772491	6.711847782	3.040067673
6.561181068	4.811152458	5.447054386
4.638926506	4.835194588	7.908195972
4.658352375	6.711847782	0.668217480
4.896168709	6.698466301	10.379202843
10.362502098	6.711847782	3.040067673
10.380790710	4.808238029	5.443077087
8.455976486	4.789724350	7.866667747
8.461192131	6.711847782	0.668217480
8.479111671	6.706594944	10.198441505
2.757042646	10.514687538	3.040067673
2.750353813	8.611402512	5.449160099
0.838376462	8.582281113	7.923643589
0.855622470	10.514687538	0.668217480
0.871232867	10.509406090	10.254340172
6.559772491	10.514687538	3.040067673
6.559772491	8.613267899	5.474937439
4.630492687	8.566788673	7.917126656
4.658352375	10.514687538	0.668217480
4.646914005	10.476099968	10.295234680
10.362502098	10.514687538	3.040067673
10.378965378	8.615572929	5.441610336
8.463705063	8.631688118	7.874871254
8.461192131	10.514687538	0.668217480
8.466629982	10.523473740	10.265063286

G6

3.487140417	6.115472794	13.944231033
3.337726831	3.625149965	13.863124847
2.441654205	6.623816490	11.903494835
2.426342010	2.970681190	11.886137962
5.065596104	6.919274807	13.726165771
4.860306263	2.694139004	13.819619179
6.284758568	5.402069092	14.983028412
5.983858585	4.740197659	12.776206970
4.404161453	6.127461433	13.331614494
4.299254417	3.508825302	13.335785866
5.143927574	4.783940315	13.492163658
2.769130945	2.910247087	5.038816929
2.757042646	1.007697463	3.423067570
2.757042646	2.909117460	1.051217556
1.420453787	2.911564827	11.855580330
2.769701958	1.061011434	7.492852211
0.855622470	1.007697463	0.285207510
0.625126004	1.006047249	9.906573296
0.857124150	2.917514086	8.403333664
0.878084958	1.009122610	5.899397373
0.855622470	2.909117460	2.657057762
6.550482750	2.906280994	5.039745808
6.559772491	1.007697463	3.423067570
6.559772491	2.909117460	1.051217556
6.720122337	2.903573990	11.017506599
6.558607101	0.993177712	7.451142788
4.658352375	1.007697463	0.285207510
4.591966152	1.059136391	9.964769363
4.762619019	2.918375015	8.375753403
4.651970387	1.010489941	5.911643505
4.658352375	2.909117460	2.657057762
10.397079468	2.907269955	5.039001465
10.362502098	1.007697463	3.423067570
10.362502098	2.909117460	1.051217556
10.036394119	2.890955687	11.016366959
10.381732941	1.008264422	7.444232941
8.461192131	1.007697463	0.285207510
8.309630394	1.008623242	9.814351082
8.522387505	2.902291536	8.237574577
8.466305733	0.996720374	5.847212791
8.461192131	2.909117460	2.657057762
2.771360159	6.710395336	5.038916111
2.757042646	4.810537338	3.423067570
2.757042646	6.711847782	1.051217556
1.437429547	6.695798874	11.854639053
2.774752617	4.809107780	7.510587215
0.855622470	4.810537338	0.285207510
0.552913427	4.808368206	9.904776573
0.858234763	6.700028896	8.407077789
0.884084046	4.811900139	5.903375149

0.855622470	6.711847782	2.657057762
6.553316116	6.715925694	5.043582916
6.559772491	4.810537338	3.423067570
6.559772491	6.711847782	1.051217556
6.727572441	6.720536709	11.022914886
6.579057693	4.809338093	7.426058769
4.658352375	4.810537338	0.285207510
4.813798428	4.809228420	9.902050018
4.765476227	6.698968887	8.374619484
4.656023979	4.809990406	5.890537262
4.658352375	6.711847782	2.657057762
10.395690918	6.713598251	5.039316654
10.362502098	4.810537338	3.423067570
10.362502098	6.711847782	1.051217556
10.044258118	6.725480080	11.019800186
10.380575180	4.808789730	7.433424473
8.461192131	4.810537338	0.285207510
8.353425980	4.809546947	9.794856071
8.526774406	6.715975761	8.240236282
8.477272987	4.808709145	5.825386524
8.461192131	6.711847782	2.657057762
2.772830963	10.513538361	5.045669079
2.757042646	8.613267899	3.423067570
2.757042646	10.514687538	1.051217556
2.628008842	10.513634682	10.800805092
2.771125078	8.557508469	7.496292591
0.855622470	8.613267899	0.285207510
0.631066680	8.611091614	9.911059380
0.987542808	10.513163567	8.341349602
0.882690609	8.609415054	5.901187897
0.855622470	10.514687538	2.657057762
6.542416096	10.511784554	5.042471409
6.559772491	8.613267899	3.423067570
6.559772491	10.514687538	1.051217556
6.442885876	10.512099266	10.852784157
6.565888882	8.623789787	7.461867809
4.658352375	8.613267899	0.285207510
4.591888428	8.556910515	9.967323303
4.718691826	10.512848854	8.381609917
4.656173706	8.608624458	5.919662476
4.658352375	10.514687538	2.657057762
10.399415970	10.513953209	5.039831638
10.362502098	8.613267899	3.423067570
10.362502098	10.514687538	1.051217556
10.151995659	10.515571594	10.851413727
10.388215065	8.611103058	7.447345734
8.461192131	8.613267899	0.285207510
8.321071625	8.610838890	9.818788528
8.566986084	10.514777184	8.275899887
8.474554062	8.624223709	5.854547024
8.461192131	10.514687538	2.657057762

4.039878368	6.468496323	11.992319107
4.015927792	3.142945051	11.983637810
5.615034103	4.709527493	14.847434044
2.757042646	2.909117460	3.040067673
2.764290571	1.008046985	5.454429150
0.854849160	1.044837236	7.938461304
0.855622470	2.909117460	0.668217480
0.372396022	2.907165766	10.402546883
6.559772491	2.909117460	3.040067673
6.541567326	1.003094673	5.447886467
4.642933369	1.051609278	7.945835114
4.658352375	2.909117460	0.668217480
5.023589134	2.921766996	10.423426628
10.362502098	2.909117460	3.040067673
10.394162178	1.006409526	5.446969986
8.455670357	0.984958351	7.830249786
8.461192131	2.909117460	0.668217480
8.412322044	2.900322199	10.173086166
2.757042646	6.711847782	3.040067673
2.764939785	4.809566021	5.451703072
0.870466590	4.809017658	7.945919514
0.855622470	6.711847782	0.668217480
0.382391453	6.708057404	10.406091690
6.559772491	6.711847782	3.040067673
6.549915314	4.810999393	5.435489655
4.673080921	4.807745457	7.915884018
4.658352375	6.711847782	0.668217480
5.032751083	6.694612026	10.419585228
10.362502098	6.711847782	3.040067673
10.403155327	4.809938431	5.438853741
8.478509903	4.808782578	7.803835869
8.461192131	6.711847782	0.668217480
8.420646667	6.716451168	10.175107956
2.757042646	10.514687538	3.040067673
2.772910357	8.611044884	5.454800606
0.859470427	8.573425293	7.943053246
0.855622470	10.514687538	0.668217480
0.909496784	10.513905525	10.264749527
6.559772491	10.514687538	3.040067673
6.559772491	8.613267899	5.474937439
4.641992569	8.565411568	7.948094368
4.658352375	10.514687538	0.668217480
4.725513458	10.512899399	10.290946007
10.362502098	10.514687538	3.040067673
10.405467987	8.613580704	5.446672916
8.469012260	8.635279655	7.840216160
8.461192131	10.514687538	0.668217480
8.479927063	10.516135216	10.245438576

CONTCAR R110

H 8 C 3 O 99 Ti 48

1.0

11.8640003204	0.0000000000	0.0000000000
0.0000000000	13.1528997421	0.0000000000
0.0000000000	0.0000000000	29.1483993530

H	C	O	Ti
8	3	99	48

G1

6.612384796	7.165732384	12.629879951
6.578532696	7.524497032	9.926907539
8.752571106	6.217486382	13.479660034
8.421349525	6.610641956	10.956965446
6.413267136	5.231364727	14.726473808
5.012085438	5.631027699	11.931227684
8.124770164	4.560896873	13.221415520
7.688590050	4.972312927	10.760206223
6.870702267	6.114307404	12.402167320
7.841917515	5.602374554	13.467969894
7.491149426	6.022061348	11.013349533
2.181430340	0.590642512	1.098609924
2.181430340	3.992062569	4.608569622
2.184775352	0.562636435	8.072758675
2.181430340	4.719442368	1.098609924
2.181430340	1.318022490	4.608569622
2.161184788	4.690774441	8.039414406
0.698430002	2.655042410	0.040089998
0.697756827	5.939419270	3.268819094
0.697512865	2.620595932	6.545552731
0.698430002	2.655042410	2.617779970
0.697327733	5.942743778	5.861067295
0.696379066	2.625778437	9.120933533
5.147429943	0.590642512	1.098609924
5.147429943	3.992062569	4.608569622
5.140756607	0.569559753	8.071905136
5.147429943	4.719442368	1.098609924
5.147429943	1.318022490	4.608569622
5.143193245	4.707162380	8.055499077
3.664430141	2.655042410	0.040089998
3.674214602	5.945084095	3.271848679
3.665916204	2.634454012	6.545710564
3.664430141	2.655042410	2.617779970
3.648887396	5.913410664	5.860555172
3.662311792	2.645425320	9.118630409
8.113430977	0.590642512	1.098609924
8.113430977	3.992062569	4.608569622
8.116349220	0.570214510	8.071316719
8.113430977	4.719442368	1.098609924
8.113430977	1.318022490	4.608569622
8.105556488	4.715516567	8.089685440

6.630430698	2.655042410	0.040089998
6.629055023	5.941916466	3.282560825
6.632057667	2.630748034	6.546767235
6.630430698	2.655042410	2.617779970
6.630573273	5.940349579	5.909413338
6.629753113	2.642492294	9.119538307
11.079429626	0.590642512	1.098609924
11.079429626	3.992062569	4.608569622
11.070365906	0.563261986	8.070140839
11.079429626	4.719442368	1.098609924
11.079429626	1.318022490	4.608569622
11.096261024	4.690753460	8.040171623
9.596429825	2.655042410	0.040089998
9.585771561	5.945520401	3.271091938
9.591122627	2.642218590	6.547370911
9.596429825	2.655042410	2.617779970
9.608316422	5.908205986	5.858171940
9.593380928	2.645540953	9.120921135
2.181430340	7.167091846	1.098609924
2.181430340	10.568512917	4.608569622
2.181974173	7.141099453	8.061314583
2.181430340	11.295892715	1.098609924
2.181430340	7.894472122	4.608569622
2.176792383	11.270123482	8.065805435
0.698430002	9.231492043	0.040089998
0.698148370	12.520494461	3.279690981
0.700750709	9.207007408	6.556398869
0.698430002	9.231492043	2.617779970
0.697825789	12.514280319	5.888097763
0.690984070	9.207167625	9.129239082
5.147429943	7.167091846	1.098609924
5.147429943	10.568512917	4.608569622
5.085072517	7.177382469	7.915046215
5.147429943	11.295892715	1.098609924
5.147429943	7.894472122	4.608569622
5.157115459	11.275358200	8.092435837
3.664430141	9.231492043	0.040089998
3.664189577	12.520491600	3.279732466
3.666248560	9.239499092	6.551023483
3.664430141	9.231492043	2.617779970
3.665248871	12.510909081	5.889596462
3.667979956	9.202078819	9.120948792
8.113430977	7.167091846	1.098609924
8.113430977	10.568512917	4.608569622
8.176720619	7.177622795	7.904839039
8.113430977	11.295892715	1.098609924
8.113430977	7.894472122	4.608569622
8.098500252	11.275021553	8.093194008
6.630430698	9.231492043	0.040089998
6.630208015	12.520078659	3.279629230
6.633849621	9.276514053	6.548132420

6.630430698	9.231492043	2.617779970
6.629944801	12.517107010	5.888941765
6.625119686	9.036138535	9.127230644
11.079429626	7.167091846	1.098609924
11.079429626	10.568512917	4.608569622
11.070016861	7.139985085	8.061573982
11.079429626	11.295892715	1.098609924
11.079429626	7.894472122	4.608569622
11.078438759	11.270350456	8.069448471
9.596429825	9.231492043	0.040089998
9.596413612	12.520508766	3.279556274
9.602477074	9.242329597	6.550847530
9.596429825	9.231492043	2.617779970
9.594281197	12.510334015	5.889447212
9.578756332	9.196600914	9.121805191
7.275109768	5.686800480	14.769076347
6.555795670	6.510336399	10.003138542
5.708996773	5.282812119	12.513546944
2.181430340	2.655042410	1.153530002
2.191436529	6.032901287	4.554637432
2.176923752	2.634603500	8.001773834
0.698430002	5.943272591	1.403370023
0.698494613	2.740315199	4.585622311
0.694736123	5.929634094	7.722984791
5.147429943	2.655042410	1.153530002
5.181767941	6.022003174	4.631316662
5.144096851	2.641586542	8.003044128
3.664430141	5.943272591	1.403370023
3.664855480	2.737271309	4.583698750
3.654974699	5.899172306	7.720125198
8.113430977	2.655042410	1.153530002
8.076544762	6.023808956	4.628723145
8.111548424	2.641453028	7.999119759
6.630430698	5.943272591	1.403370023
6.630424976	2.733279943	4.585335255
6.620508671	5.980370998	7.943333626
11.079429626	2.655042410	1.153530002
11.067687988	6.034498692	4.554720402
11.078228951	2.636386395	8.004036903
9.596429825	5.943272591	1.403370023
9.596235275	2.739939928	4.581848145
9.597904205	5.900577545	7.716970444
2.181430340	9.231492043	1.153530002
2.181198359	12.611206055	4.586284161
2.163836956	9.195904732	8.008913040
0.698430002	12.519721985	1.403370023
0.697268546	9.314091682	4.585050583
0.695642292	12.499905586	7.758258343
5.147429943	9.231492043	1.153530002
5.147141457	12.606575012	4.586518764
5.115608215	9.232316017	7.972349644

3.664430141	12.519721985	1.403370023
3.667709112	9.304474831	4.571881771
3.658838749	12.488840103	7.760097980
8.113430977	9.231492043	1.153530002
8.113195419	12.607744217	4.586139679
8.115114212	9.225897789	7.972229481
6.630430698	12.519721985	1.403370023
6.628871441	9.302522659	4.582118988
6.628836632	12.522484779	7.759369373
11.079429626	9.231492043	1.153530002
11.078782082	12.611629486	4.586547852
11.068193436	9.196811676	8.008259773
9.596429825	12.519721985	1.403370023
9.590144157	9.306576729	4.574100494
9.596710205	12.487153053	7.760055542

G2

6.178689480	8.610016823	12.495786667
6.690036774	7.591969013	10.053214073
8.555401802	7.134043694	12.019264221
7.427443981	6.856000900	13.373932838
5.645311356	6.130843639	11.804652214
8.072061539	4.546779633	10.939398766
6.339478970	3.263287783	10.700764656
7.695597649	4.663046837	12.668275833
7.494938374	7.151475906	12.312457085
6.703148365	6.136379719	11.489377975
7.251770973	4.718698978	11.657707214
2.181430340	0.590642512	1.098609924
2.181430340	3.992062569	4.608569622
2.185446978	0.606591403	8.057556152
2.181430340	4.719442368	1.098609924
2.181430340	1.318022490	4.608569622
2.168722630	4.731390476	8.050740242
0.698430002	2.655042410	0.040089998
0.701269329	5.943546772	3.268239737
0.700556695	2.663850546	6.552358150
0.698430002	2.655042410	2.617779970
0.699609697	5.951344967	5.857840061
0.694547057	2.663754940	9.127080917
5.147429943	0.590642512	1.098609924
5.147429943	3.992062569	4.608569622
5.141311646	0.612514973	8.049322128
5.147429943	4.719442368	1.098609924
5.147429943	1.318022490	4.608569622
5.156518459	4.751920223	8.103301048
3.664430141	2.655042410	0.040089998
3.676338911	5.944013596	3.272690773
3.674043179	2.680508852	6.547930717
3.664430141	2.655042410	2.617779970
3.654101372	5.929938793	5.872042179

3.667810202	2.674285650	9.113845825
8.113430977	0.590642512	1.098609924
8.113430977	3.992062569	4.608569622
8.118618965	0.612125397	8.056543350
8.113430977	4.719442368	1.098609924
8.113430977	1.318022490	4.608569622
8.116319656	4.753067970	8.103986740
6.630430698	2.655042410	0.040089998
6.630732536	5.943057537	3.286397219
6.636341572	2.670258760	6.545583248
6.630430698	2.655042410	2.617779970
6.630560875	5.932133675	5.928481102
6.626406193	2.662842512	9.110777855
11.079429626	0.590642512	1.098609924
11.079429626	3.992062569	4.608569622
11.074563026	0.607660830	8.059078217
11.079429626	4.719442368	1.098609924
11.079429626	1.318022490	4.608569622
11.093997955	4.733465195	8.050342560
9.596429825	2.655042410	0.040089998
9.585429192	5.944101810	3.271291018
9.592566490	2.676738977	6.551517963
9.596429825	2.655042410	2.617779970
9.611805916	5.926495075	5.863653183
9.587770462	2.682137489	9.121355057
2.181430340	7.167091846	1.098609924
2.181430340	10.568512917	4.608569622
2.187456131	7.180869579	8.049997330
2.181430340	11.295892715	1.098609924
2.181430340	7.894472122	4.608569622
2.178471088	11.312567711	8.074476242
0.698430002	9.231492043	0.040089998
0.698529840	12.518307686	3.277974606
0.699438035	9.247364998	6.552861214
0.698430002	9.231492043	2.617779970
0.698342443	12.522109032	5.885432243
0.699549019	9.243727684	9.125876427
5.147429943	7.167091846	1.098609924
5.147429943	10.568512917	4.608569622
5.099901676	7.215983868	7.927888870
5.147429943	11.295892715	1.098609924
5.147429943	7.894472122	4.608569622
5.156964779	11.319833755	8.095145226
3.664430141	9.231492043	0.040089998
3.664326191	12.519210815	3.278399467
3.660262108	9.274720192	6.542781353
3.664430141	9.231492043	2.617779970
3.664908409	12.517599106	5.886298180
3.675621510	9.242378235	9.114187241
8.113430977	7.167091846	1.098609924
8.113430977	10.568512917	4.608569622

8.164224625	7.221675396	7.885429382
8.113430977	11.295892715	1.098609924
8.113430977	7.894472122	4.608569622
8.104575157	11.319568634	8.090669632
6.630430698	9.231492043	0.040089998
6.630700111	12.518502235	3.278326511
6.627078533	9.315542221	6.529705048
6.630430698	9.231492043	2.617779970
6.630678177	12.524048805	5.885757923
6.631946564	9.115251541	9.095636368
11.079429626	7.167091846	1.098609924
11.079429626	10.568512917	4.608569622
11.077182770	7.182620049	8.045880318
11.079429626	11.295892715	1.098609924
11.079429626	7.894472122	4.608569622
11.081793785	11.313318253	8.076928139
9.596429825	9.231492043	0.040089998
9.596831322	12.519037247	3.278499603
9.604121208	9.283489227	6.539788723
9.596429825	9.231492043	2.617779970
9.596242905	12.518959045	5.886909485
9.586458206	9.232986450	9.108708382
7.060843468	8.493135452	12.099398613
6.724368095	6.582057476	10.085628510
6.246299744	3.729236841	11.568350792
2.181430340	2.655042410	1.153530002
2.199614525	5.849272728	4.551474571
2.168443918	2.679291725	8.005800247
0.698430002	5.943272591	1.403370023
0.696924746	2.573765993	4.585384846
0.700360119	5.958858490	7.716328144
5.147429943	2.655042410	1.153530002
5.195878983	5.862650871	4.652488232
5.123219967	2.678541899	7.973669052
3.664430141	5.943272591	1.403370023
3.671013355	2.582944870	4.569755554
3.665379524	5.948445320	7.730179310
8.113430977	2.655042410	1.153530002
8.066050529	5.862485409	4.649921417
8.117957115	2.677620411	7.981050968
6.630430698	5.943272591	1.403370023
6.629050732	2.589101791	4.581942081
6.636231422	6.015497208	8.054430008
11.079429626	2.655042410	1.153530002
11.066024780	5.849484921	4.546628952
11.075347900	2.682523727	8.005724907
9.596429825	5.943272591	1.403370023
9.587354660	2.583388805	4.573109150
9.600365639	5.956384659	7.718481064
2.181430340	9.231492043	1.153530002
2.181413174	12.431184769	4.584304333

2.179374456	9.236328125	8.005057335
0.698430002	12.519721985	1.403370023
0.698104858	9.149847031	4.582831383
0.698378742	12.539860725	7.755129337
5.147429943	9.231492043	1.153530002
5.147489548	12.429566383	4.584435463
5.133638859	9.270167351	7.963245392
3.664430141	12.519721985	1.403370023
3.663418531	9.145420074	4.572476387
3.659781933	12.526875496	7.755680561
8.113430977	9.231492043	1.153530002
8.113669395	12.430259705	4.584837437
8.130319595	9.275054932	7.961585522
6.630430698	12.519721985	1.403370023
6.630479813	9.155094147	4.581547260
6.629943371	12.559206963	7.754377842
11.079429626	9.231492043	1.153530002
11.079068184	12.431013107	4.584734917
11.085551262	9.240058899	8.002754211
9.596429825	12.519721985	1.403370023
9.596948624	9.146423340	4.573050976
9.600963593	12.529794693	7.756794453

G3

5.860924244	7.039873123	11.696039200
7.270170212	8.369013786	10.592214584
7.170415401	6.637104988	12.841183662
6.511333942	3.666992426	10.963768959
6.851606369	5.629274368	9.973721504
9.013895988	7.069791317	10.423525810
9.169996262	5.255987644	11.957726479
8.939142227	4.205072403	10.528250694
6.925302029	6.776055336	11.777042389
7.220391273	5.478855610	11.019696236
8.730151176	5.194463730	10.950433731
2.181430340	0.590642512	1.098609924
2.181430340	3.992062569	4.608569622
2.179027557	0.602623522	8.061810493
2.181430340	4.719442368	1.098609924
2.181430340	1.318022490	4.608569622
2.188020229	4.730978012	8.055890083
0.698430002	2.655042410	0.040089998
0.688832581	5.944632053	3.272461653
0.699974358	2.669085979	6.551992893
0.698430002	2.655042410	2.617779970
0.705783606	5.940640926	5.877283573
0.692073703	2.670859814	9.122026443
5.147429943	0.590642512	1.098609924
5.147429943	3.992062569	4.608569622
5.151153088	0.603734910	8.070742607
5.147429943	4.719442368	1.098609924

5.147429943	1.318022490	4.608569622
5.129642963	4.727451801	8.028526306
3.664430141	2.655042410	0.040089998
3.659046650	5.944450855	3.267947912
3.669301748	2.662299871	6.553472996
3.664430141	2.655042410	2.617779970
3.662035704	5.952743053	5.856599331
3.665339231	2.663368225	9.125456810
8.113430977	0.590642512	1.098609924
8.113430977	3.992062569	4.608569622
8.103528023	0.605111957	8.070453644
8.113430977	4.719442368	1.098609924
8.113430977	1.318022490	4.608569622
8.115805626	4.725180626	7.989021301
6.630430698	2.655042410	0.040089998
6.640881538	5.943797112	3.268652916
6.628523827	2.645996809	6.551117897
6.630430698	2.655042410	2.617779970
6.614174366	5.941468239	5.853652477
6.634530544	2.722282887	9.136757851
11.079429626	0.590642512	1.098609924
11.079429626	3.992062569	4.608569622
11.083579063	0.606356204	8.062030792
11.079429626	4.719442368	1.098609924
11.079429626	1.318022490	4.608569622
11.081308365	4.734869480	8.066902161
9.596429825	2.655042410	0.040089998
9.596947670	5.943411827	3.282745361
9.595824242	2.648330212	6.545743942
9.596429825	2.655042410	2.617779970
9.599934578	5.945450783	5.913936138
9.583335876	2.670216560	9.109511375
2.181430340	7.167091846	1.098609924
2.181430340	10.568512917	4.608569622
2.177122831	7.180855274	8.051099777
2.181430340	11.295892715	1.098609924
2.181430340	7.894472122	4.608569622
2.185564518	11.308666229	8.073244095
0.698430002	9.231492043	0.040089998
0.699102461	12.518937111	3.279082060
0.698437393	9.263642311	6.550569534
0.698430002	9.231492043	2.617779970
0.697801709	12.518486977	5.887628555
0.699585915	9.249232292	9.122649193
5.147429943	7.167091846	1.098609924
5.147429943	10.568512917	4.608569622
5.145825863	7.178304195	8.044669151
5.147429943	11.295892715	1.098609924
5.147429943	7.894472122	4.608569622
5.147731781	11.307412148	8.082194328
3.664430141	9.231492043	0.040089998

3.663467646	12.518529892	3.278571606
3.667023659	9.243935585	6.557419300
3.664430141	9.231492043	2.617779970
3.665365219	12.521875381	5.887886524
3.673622370	9.238860130	9.128496170
8.113430977	7.167091846	1.098609924
8.113430977	10.568512917	4.608569622
8.070382118	7.213205338	7.917444706
8.113430977	11.295892715	1.098609924
8.113430977	7.894472122	4.608569622
8.116950989	11.315941811	8.079689980
6.630430698	9.231492043	0.040089998
6.630129814	12.518968582	3.278387070
6.615198612	9.284088135	6.543321609
6.630430698	9.231492043	2.617779970
6.629844189	12.519630432	5.885660648
6.652472973	9.203802109	9.129556656
11.079429626	7.167091846	1.098609924
11.079429626	10.568512917	4.608569622
11.111836433	7.203788280	7.998976707
11.079429626	11.295892715	1.098609924
11.079429626	7.894472122	4.608569622
11.070698738	11.316113472	8.083510399
9.596429825	9.231492043	0.040089998
9.597033501	12.518071175	3.278622627
9.590922356	9.287126541	6.533949375
9.596429825	9.231492043	2.617779970
9.595985413	12.525767326	5.886674404
9.584350586	9.189993858	9.095457077
7.765577316	7.831640720	11.267383575
6.529976368	4.406907558	11.614790916
9.413156509	6.184022427	10.111099243
2.181430340	2.655042410	1.153530002
2.162412882	5.849678993	4.554885864
2.173828840	2.675334454	8.006014824
0.698430002	5.943272591	1.403370023
0.689322889	2.586539745	4.579333782
0.697644770	5.939622402	7.737261772
5.147429943	2.655042410	1.153530002
5.160425186	5.849757195	4.545123100
5.133154392	2.673970699	8.000966072
3.664430141	5.943272591	1.403370023
3.664432526	2.571483135	4.583226681
3.662882090	5.962805748	7.714574337
8.113430977	2.655042410	1.153530002
8.158991814	5.866342068	4.646272182
8.104668617	2.661702871	7.967859268
6.630430698	5.943272591	1.403370023
6.637155533	2.584822893	4.575040817
6.630446911	5.941635132	7.715497971
11.079429626	2.655042410	1.153530002

11.032916069	5.866082191	4.647646904
11.076888084	2.676834345	7.995049953
9.596429825	5.943272591	1.403370023
9.595600128	2.592348099	4.576619148
9.589777946	6.006506443	8.033838272
2.181430340	9.231492043	1.153530002
2.181573153	12.433422089	4.585675716
2.192353725	9.230571747	8.005866051
0.698430002	12.519721985	1.403370023
0.699844718	9.148944855	4.580336571
0.702127934	12.522762299	7.757249832
5.147429943	9.231492043	1.153530002
5.146883965	12.431205750	4.584586143
5.151927471	9.233419418	8.005577087
3.664430141	12.519721985	1.403370023
3.664947033	9.147361755	4.581988811
3.663740158	12.536836624	7.758450985
8.113430977	9.231492043	1.153530002
8.113101959	12.434312820	4.584596157
8.131867409	9.275150299	7.940419674
6.630430698	12.519721985	1.403370023
6.630109310	9.148264885	4.576741219
6.623564720	12.523297310	7.754914761
11.079429626	9.231492043	1.153530002
11.079832077	12.435265541	4.585100651
11.099056244	9.260024071	7.994679928
9.596429825	12.519721985	1.403370023
9.598367691	9.156492233	4.569136143
9.598362923	12.556253433	7.755494118

G4

7.319637775	6.721579075	10.352379799
3.586490154	4.225761890	9.801084518
7.012414455	8.664268494	10.927845955
4.930161953	7.407946587	10.233677864
6.407907486	6.573078632	12.794948578
5.032302856	4.590706825	11.365870476
5.342841148	7.987182617	12.511590004
3.841628790	5.792509556	11.967324257
5.446477890	6.633214951	10.821317673
6.076943398	7.309588432	12.047989845
4.483780384	5.511418819	11.116582870
2.181430340	0.590642512	1.098609924
2.181430340	3.992062569	4.608569622
2.195372820	0.598032475	8.091976166
2.181430340	4.719442368	1.098609924
2.181430340	1.318022490	4.608569622
2.118428230	4.692070961	7.888803959
0.698430002	2.655042410	0.040089998
0.709907472	5.942284584	3.265464544
0.697807908	2.632444382	6.550183296

0.698430002	2.655042410	2.617779970
0.685571373	5.976047993	5.845638275
0.713425934	2.671784401	9.117719650
5.147429943	0.590642512	1.098609924
5.147429943	3.992062569	4.608569622
5.138369560	0.600788295	8.100495338
5.147429943	4.719442368	1.098609924
5.147429943	1.318022490	4.608569622
5.211603642	4.693736553	7.870528698
3.664430141	2.655042410	0.040089998
3.676502228	5.946163654	3.280550003
3.665943146	2.590462685	6.542100906
3.664430141	2.655042410	2.617779970
3.651404142	5.936946392	5.906756878
3.664492369	2.835919142	9.115990639
8.113430977	0.590642512	1.098609924
8.113430977	3.992062569	4.608569622
8.120281219	0.607022464	8.073468208
8.113430977	4.719442368	1.098609924
8.113430977	1.318022490	4.608569622
8.139352798	4.727714062	8.025465965
6.630430698	2.655042410	0.040089998
6.617236614	5.941041470	3.277432680
6.634606361	2.624377966	6.551519871
6.630430698	2.655042410	2.617779970
6.649054527	5.973267555	5.897563457
6.621214867	2.685000896	9.116051674
11.079429626	0.590642512	1.098609924
11.079429626	3.992062569	4.608569622
11.083300591	0.603472829	8.072488785
11.079429626	4.719442368	1.098609924
11.079429626	1.318022490	4.608569622
11.093487740	4.734282494	8.047476768
9.596429825	2.655042410	0.040089998
9.586900711	5.948143959	3.265117168
9.602772713	2.662267923	6.558618546
9.596429825	2.655042410	2.617779970
9.605805397	5.945604324	5.845738411
9.599137306	2.668378592	9.132823944
2.181430340	7.167091846	1.098609924
2.181430340	10.568512917	4.608569622
2.183181047	7.168150425	8.065073013
2.181430340	11.295892715	1.098609924
2.181430340	7.894472122	4.608569622
2.184327841	11.305345535	8.081604004
0.698430002	9.231492043	0.040089998
0.699255347	12.518805504	3.279258251
0.700438440	9.244363785	6.557003021
0.698430002	9.231492043	2.617779970
0.701314569	12.523190498	5.891016960
0.709338903	9.241447449	9.128219604

5.147429943	7.167091846	1.098609924
5.147429943	10.568512917	4.608569622
5.092847347	7.185621262	7.958333015
5.147429943	11.295892715	1.098609924
5.147429943	7.894472122	4.608569622
5.159560204	11.310351372	8.078037262
3.664430141	9.231492043	0.040089998
3.664920092	12.518902779	3.279180050
3.661328793	9.274122238	6.548167229
3.664430141	9.231492043	2.617779970
3.666310787	12.519616127	5.889441490
3.689060211	9.226017952	9.118801117
8.113430977	7.167091846	1.098609924
8.113430977	10.568512917	4.608569622
8.166356087	7.200892925	7.995577335
8.113430977	11.295892715	1.098609924
8.113430977	7.894472122	4.608569622
8.109047890	11.316472054	8.079281807
6.630430698	9.231492043	0.040089998
6.631604195	12.518271446	3.278736115
6.626682758	9.270614624	6.544851780
6.630430698	9.231492043	2.617779970
6.630831718	12.527418137	5.888781071
6.633966446	9.197667122	9.126586914
11.079429626	7.167091846	1.098609924
11.079429626	10.568512917	4.608569622
11.071737289	7.190330505	8.035525322
11.079429626	11.295892715	1.098609924
11.079429626	7.894472122	4.608569622
11.086130142	11.310976028	8.073113441
9.596429825	9.231492043	0.040089998
9.597830772	12.518073082	3.278975010
9.592086792	9.270669937	6.549195766
9.596429825	9.231492043	2.617779970
9.597399712	12.523286819	5.888435364
9.595455170	9.250664711	9.121068001
6.572712898	6.133489132	10.031466484
7.257963657	7.997716904	11.622059822
3.612373352	5.247724056	9.985967636
2.181430340	2.655042410	1.153530002
2.227841854	5.862719059	4.604401112
2.155486345	2.639818668	7.954875946
0.698430002	5.943272591	1.403370023
0.702570081	2.579526901	4.566398621
0.694563925	5.975661278	7.699989796
5.147429943	2.655042410	1.153530002
5.145643234	5.873394012	4.685048580
5.174200058	2.645836592	7.953946114
3.664430141	5.943272591	1.403370023
3.668877363	2.596027613	4.579336166
3.651956320	5.886256695	7.920029163

8.113430977	2.655042410	1.153530002
8.069285393	5.863765717	4.596335888
8.122460365	2.687041283	8.005452156
6.630430698	5.943272591	1.403370023
6.625980377	2.592274666	4.565463543
6.625011444	5.978697300	7.896381855
11.079429626	2.655042410	1.153530002
11.081243515	5.850938797	4.534957886
11.076148033	2.687219381	8.008683205
9.596429825	5.943272591	1.403370023
9.589996338	2.581945419	4.583050251
9.601999283	5.948173046	7.698379517
2.181430340	9.231492043	1.153530002
2.182438374	12.439606667	4.586266994
2.196885347	9.226604462	8.006587982
0.698430002	12.519721985	1.403370023
0.699450612	9.149643898	4.581502438
0.697201431	12.530622482	7.760668755
5.147429943	9.231492043	1.153530002
5.148644447	12.441047668	4.584940910
5.158157349	9.255565643	7.958775997
3.664430141	12.519721985	1.403370023
3.663012028	9.156160355	4.573336124
3.665346861	12.511580467	7.757662296
8.113430977	9.231492043	1.153530002
8.115321159	12.433091164	4.584937096
8.146696091	9.252671242	7.974762440
6.630430698	12.519721985	1.403370023
6.629432678	9.154031754	4.572210312
6.639089584	12.549356461	7.756909847
11.079429626	9.231492043	1.153530002
11.081047058	12.434366226	4.586219311
11.099136353	9.232921600	8.005969048
9.596429825	12.519721985	1.403370023
9.598698616	9.144945145	4.572996140
9.602624893	12.537129402	7.757360935

G5

5.243496895	4.724790573	12.034740448
4.355526924	5.964034557	13.792362213
7.149000168	6.381177425	11.959670067
2.906924009	5.754480839	11.873696327
6.658428192	3.348518133	9.914708138
3.649023056	4.401945114	9.993953705
6.104723454	7.513549805	11.051215172
3.630361080	7.151249409	11.024657249
5.073047638	5.817093849	11.956523895
6.290894985	6.448953629	11.271157265
3.744257689	6.078332424	11.232526779
2.181430340	0.590642512	1.098609924
2.181430340	3.992062569	4.608569622

2.198050737	0.603967488	8.104902267
2.181430340	4.719442368	1.098609924
2.181430340	1.318022490	4.608569622
2.122587681	4.676660538	7.888125420
0.698430002	2.655042410	0.040089998
0.712517381	5.942995071	3.261387110
0.688891292	2.620957375	6.589800835
0.698430002	2.655042410	2.617779970
0.688796043	5.968235016	5.830445290
0.715855241	2.669418573	9.184596062
5.147429943	0.590642512	1.098609924
5.147429943	3.992062569	4.608569622
5.143027782	0.616499126	8.090325356
5.147429943	4.719442368	1.098609924
5.147429943	1.318022490	4.608569622
5.172890186	4.670346737	7.848908424
3.664430141	2.655042410	0.040089998
3.673008204	5.941552162	3.271466017
3.668589592	2.587610483	6.572943211
3.664430141	2.655042410	2.617779970
3.650220394	5.963297367	5.904139042
3.687452078	2.818858862	9.140742302
8.113430977	0.590642512	1.098609924
8.113430977	3.992062569	4.608569622
8.121547699	0.613116324	8.066957474
8.113430977	4.719442368	1.098609924
8.113430977	1.318022490	4.608569622
8.176129341	4.680984497	7.984505653
6.630430698	2.655042410	0.040089998
6.619647026	5.941726685	3.273967743
6.628427982	2.589793444	6.587569714
6.630430698	2.655042410	2.617779970
6.646950245	5.960879326	5.886631966
6.635845661	2.699002028	9.188371658
11.079429626	0.590642512	1.098609924
11.079429626	3.992062569	4.608569622
11.080955505	0.602937818	8.086792946
11.079429626	4.719442368	1.098609924
11.079429626	1.318022490	4.608569622
11.090938568	4.719580650	8.051671028
9.596429825	2.655042410	0.040089998
9.590098381	5.943377495	3.258289814
9.605564117	2.644453526	6.573179722
9.596429825	2.655042410	2.617779970
9.614353180	5.955497742	5.843891144
9.593757629	2.659816265	9.139686584
2.181430340	7.167091846	1.098609924
2.181430340	10.568512917	4.608569622
2.170875311	7.162427425	8.053397179
2.181430340	11.295892715	1.098609924
2.181430340	7.894472122	4.608569622

2.182455540	11.307703018	8.068387032
0.698430002	9.231492043	0.040089998
0.699818611	12.518482208	3.277768373
0.701772392	9.246133804	6.546718121
0.698430002	9.231492043	2.617779970
0.701367795	12.530848503	5.889046669
0.704071105	9.238644600	9.121687889
5.147429943	7.167091846	1.098609924
5.147429943	10.568512917	4.608569622
5.128630161	7.165522575	8.062459946
5.147429943	11.295892715	1.098609924
5.147429943	7.894472122	4.608569622
5.158024311	11.313782692	8.082884789
3.664430141	9.231492043	0.040089998
3.665067911	12.522585869	3.278224468
3.665487051	9.253808022	6.545687675
3.664430141	9.231492043	2.617779970
3.666523218	12.520524025	5.890904903
3.669783592	9.228956223	9.120065689
8.113430977	7.167091846	1.098609924
8.113430977	10.568512917	4.608569622
8.156982422	7.174705982	8.042523384
8.113430977	11.295892715	1.098609924
8.113430977	7.894472122	4.608569622
8.107877731	11.316841125	8.079772949
6.630430698	9.231492043	0.040089998
6.631306171	12.520019531	3.278100491
6.627329826	9.256390572	6.546621799
6.630430698	9.231492043	2.617779970
6.630338669	12.527136803	5.889810562
6.632492542	9.215254784	9.119231224
11.079429626	7.167091846	1.098609924
11.079429626	10.568512917	4.608569622
11.079951286	7.178793430	8.030755997
11.079429626	11.295892715	1.098609924
11.079429626	7.894472122	4.608569622
11.091472626	11.310117722	8.063186646
9.596429825	9.231492043	0.040089998
9.597090721	12.521201134	3.278105497
9.594645500	9.257281303	6.544686317
9.596429825	9.231492043	2.617779970
9.597590446	12.520838737	5.888511181
9.600439072	9.252367020	9.117856979
5.021602154	6.423682213	13.252185822
6.612983227	5.759133816	10.079477310
3.633883476	5.404869080	9.964240074
2.181430340	2.655042410	1.153530002
2.240896940	5.879133701	4.589899063
2.162071705	2.666447878	8.027827263
0.698430002	5.943272591	1.403370023
0.703362584	2.584406137	4.588836670

0.709688544	5.950166702	7.683087826
5.147429943	2.655042410	1.153530002
5.185067177	5.901954174	4.719707966
5.080082417	2.662230015	7.839627266
3.664430141	5.943272591	1.403370023
3.670645952	2.607876778	4.513743877
3.695423126	5.927382469	7.890389442
8.113430977	2.655042410	1.153530002
8.045045853	5.893811703	4.642556667
8.231945038	2.662756205	7.898659229
6.630430698	5.943272591	1.403370023
6.630643368	2.623494864	4.563763618
6.660880566	5.902556419	8.267719269
11.079429626	2.655042410	1.153530002
11.073264122	5.863814831	4.522696495
11.117161751	2.688018799	8.042734146
9.596429825	5.943272591	1.403370023
9.591059685	2.592624903	4.523409843
9.600210190	5.963088512	7.698345661
2.181430340	9.231492043	1.153530002
2.182862282	12.432048798	4.589473248
2.184414864	9.217950821	7.998823643
0.698430002	12.519721985	1.403370023
0.698938131	9.145555496	4.582083225
0.700842381	12.548376083	7.760885239
5.147429943	9.231492043	1.153530002
5.148266792	12.449110031	4.589241505
5.154843330	9.225123405	7.989470482
3.664430141	12.519721985	1.403370023
3.667881727	9.158490181	4.581052780
3.667217970	12.502167702	7.764049530
8.113430977	9.231492043	1.153530002
8.114651680	12.438655853	4.588617325
8.122906685	9.222730637	7.995948315
6.630430698	12.519721985	1.403370023
6.629243851	9.168853760	4.581521988
6.634552002	12.558156013	7.761710167
11.079429626	9.231492043	1.153530002
11.081254959	12.428433418	4.588025570
11.087630272	9.218295097	7.999769688
9.596429825	12.519721985	1.403370023
9.595949173	9.146701813	4.581576824
9.603387833	12.507164001	7.760805130
